# Supplementary figures and images for: Machine-learning model selection and parameter estimation from kinetic data of complex first-order reaction systems
Source: PLoS One. 2021 Aug 9;16(8):e0255675. doi: 10.1371/journal.pone.0255675 (PMC8352076; doi:10.1371/journal.pone.0255675)

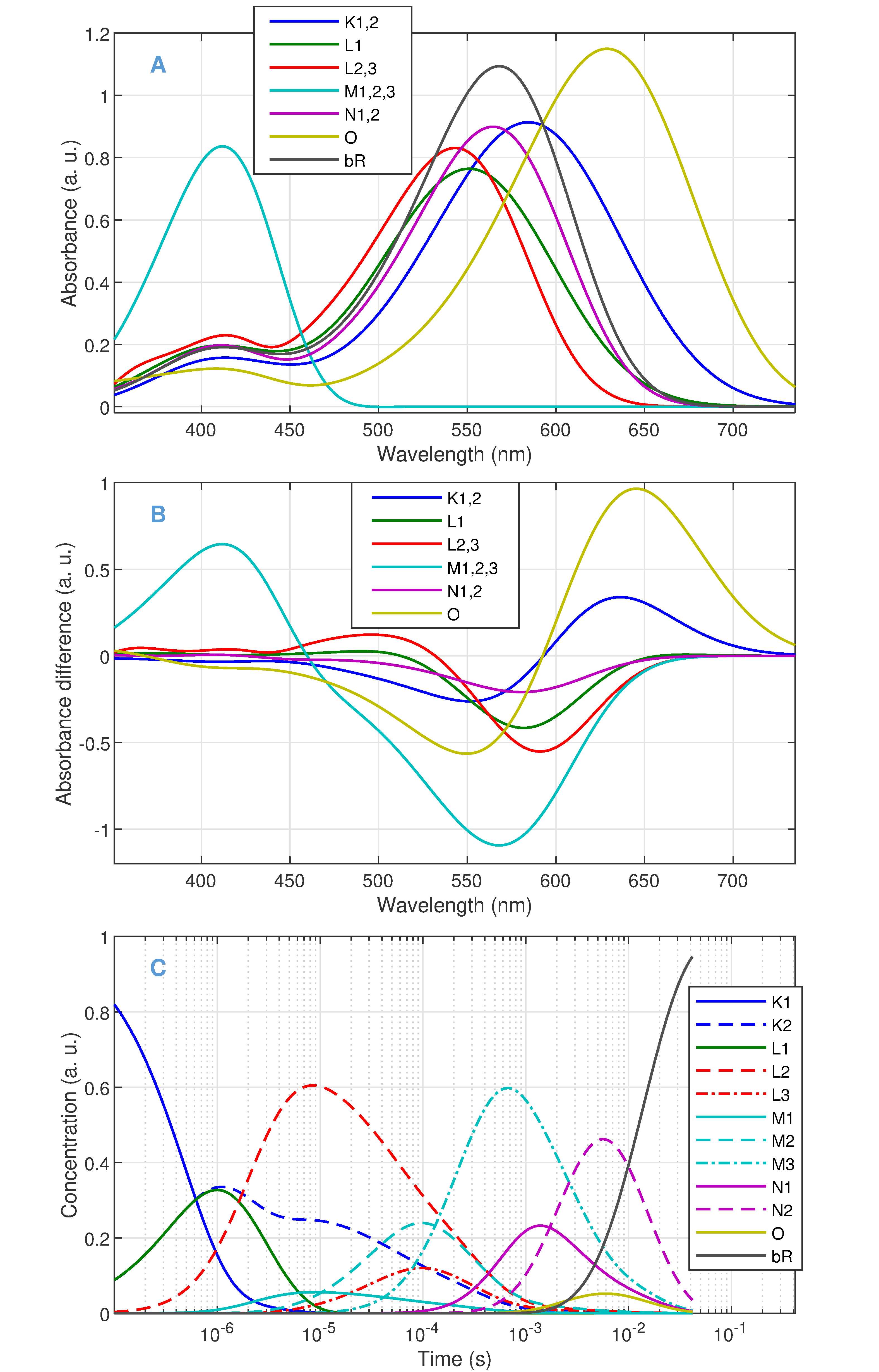

Supplement: S1 Fig — (A) Absorption spectra (B) Difference spectra obtained by subtracting the spectrum of the bR state. (C) The corresponding kinetics calculated from the microscopic rate constants. (TIF) [file pone.0255675.s001.tif]

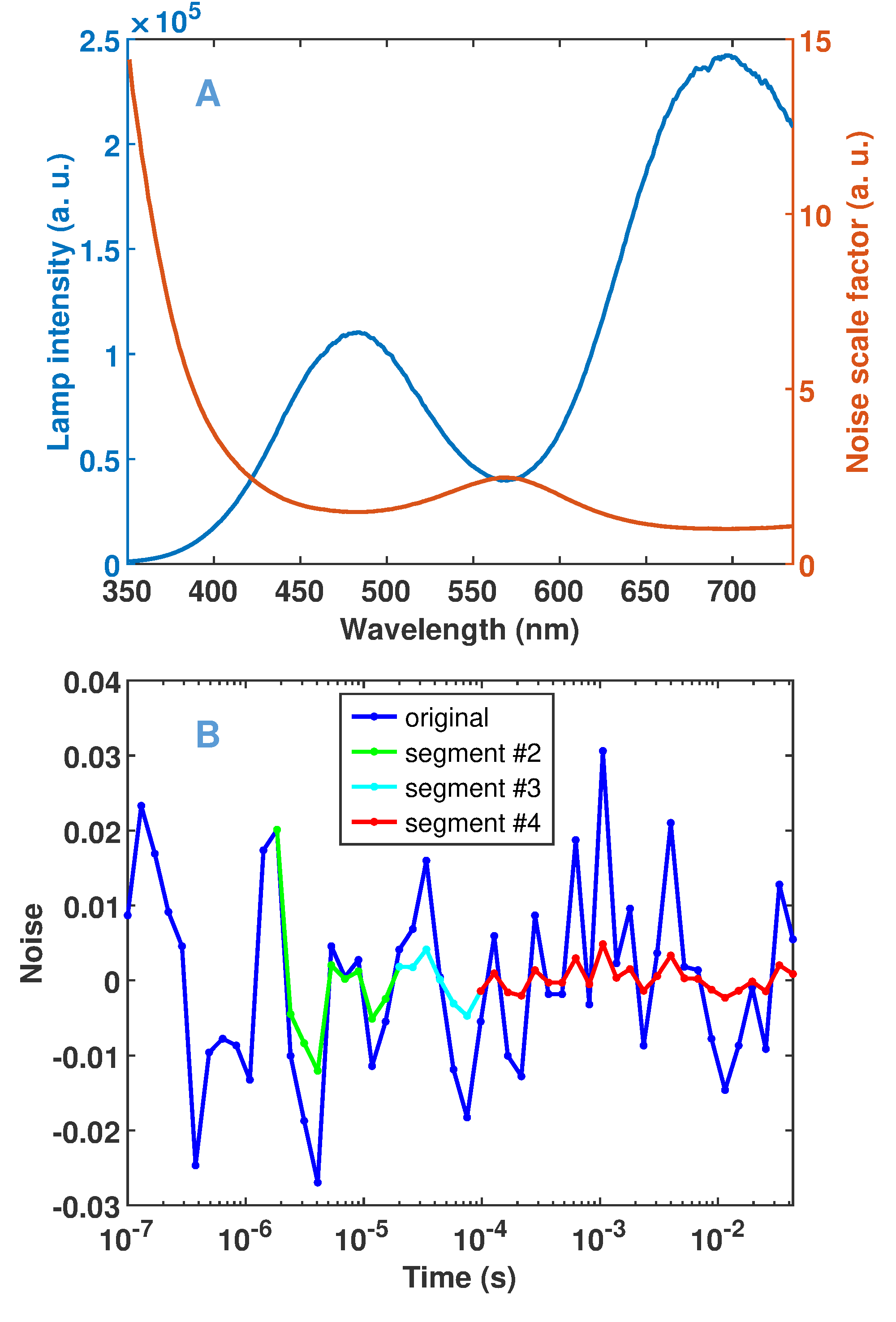

Supplement: S2 Fig — (A) Blue line: Spectral distribution of the measuring beam filtered by the bR sample. Red line: the distribution of the noise level of the blue intensity spectrum, proportional to the inverse of its square root. (B) The level of noise in the different time segments at a selected wavelength. (See Methods of the main text for details). (TIF) [file pone.0255675.s002.tif]

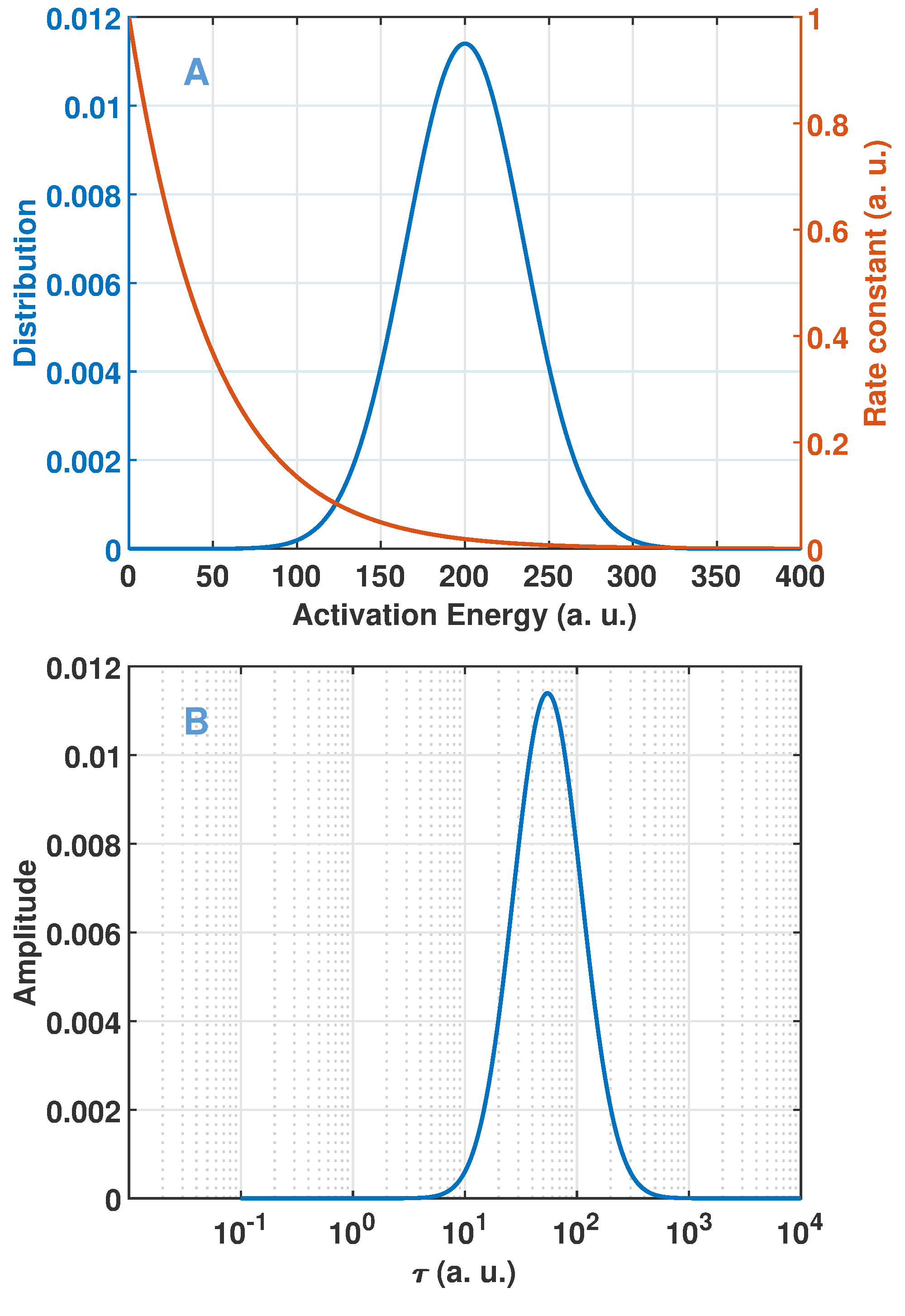

Supplement: S3 Fig — (A) The supposed rate constant (red) and distribution (blue) over the activation energy. (B) The true distribution of the time constant calculated from the data presented in (A). The resulted kinetics is presented in Fig 8 of the main text (blue line). (TIF) [file pone.0255675.s003.tif]

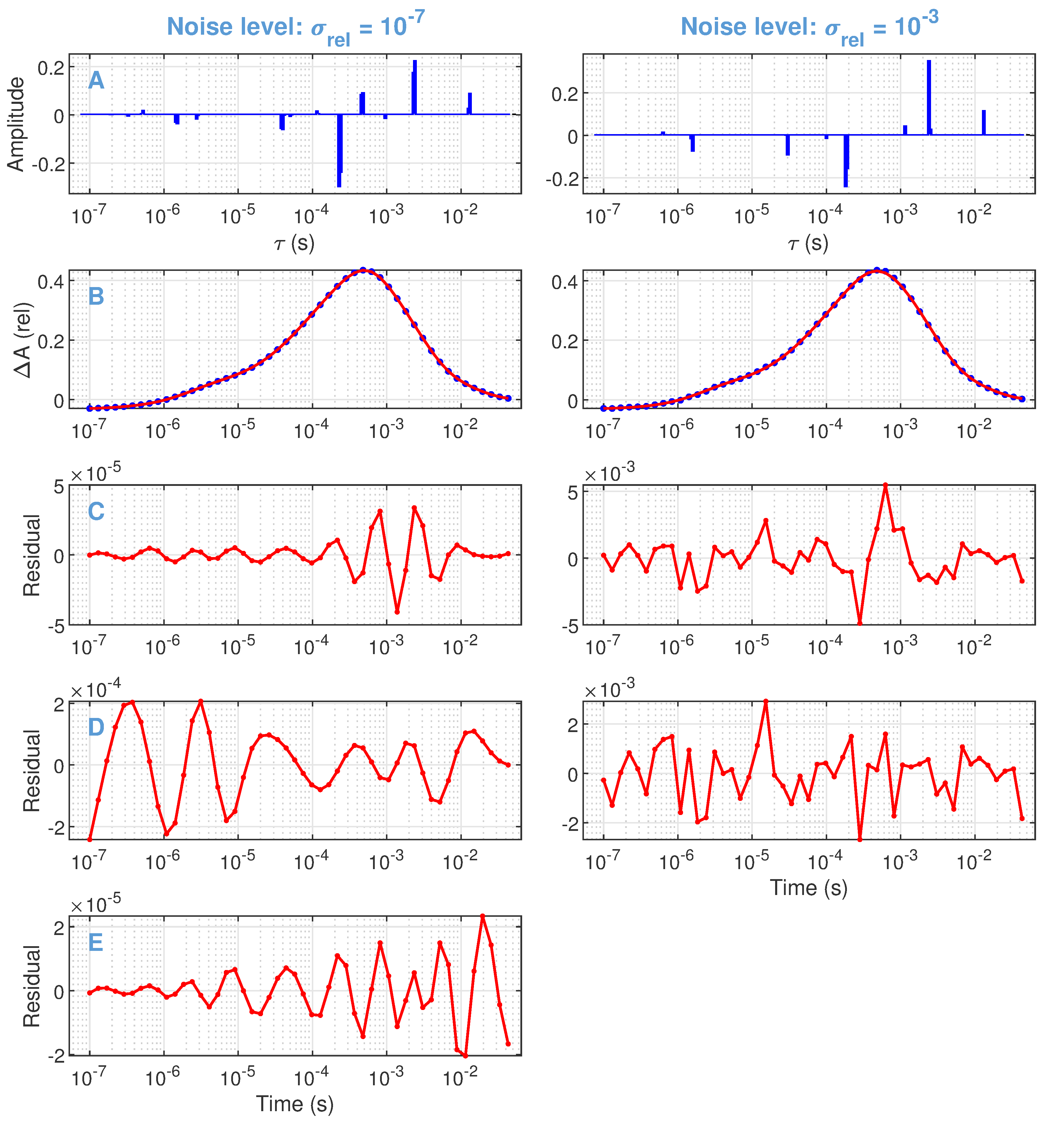

Supplement: S4 Fig — Wavelength: 416 nm. (A) Distribution over time constants. (B) Simulated kinetics (blue) and fit (red) by the distribution presented in (A). (C) Residual of the fit presented in (B). (D) Residual of the correcting exponential fit with 8 components remaining after discretization of the data, neglecting the components with amplitudes less than 5% of the maximum amplitude value. (E) Residual of the correcting exponential fit by keeping all 17 components obtained by the discretization. (TIF) [file pone.0255675.s004.tif]

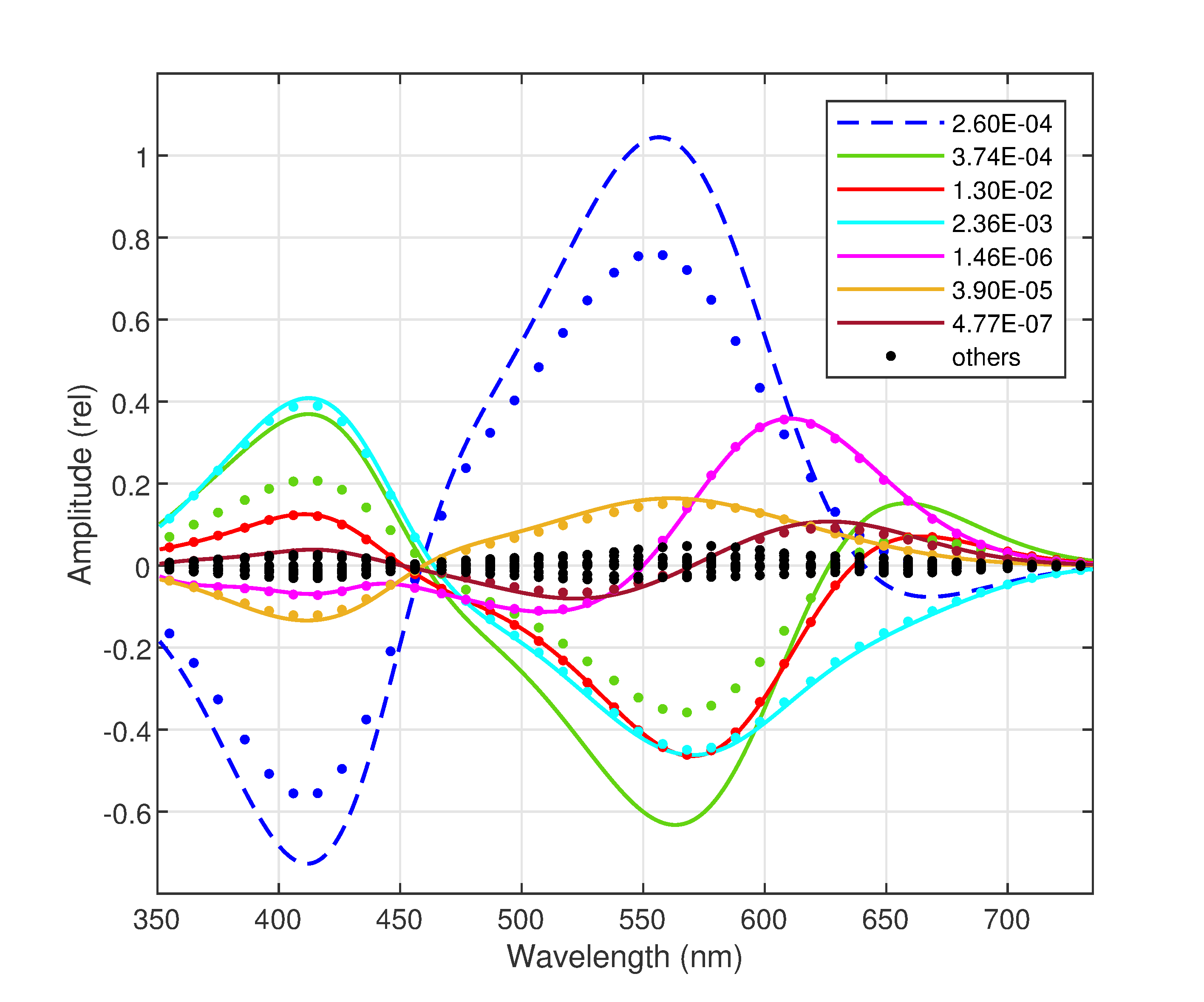

Supplement: S5 Fig — Noise level: σrel = 10−7. (TIF) [file pone.0255675.s005.tif]

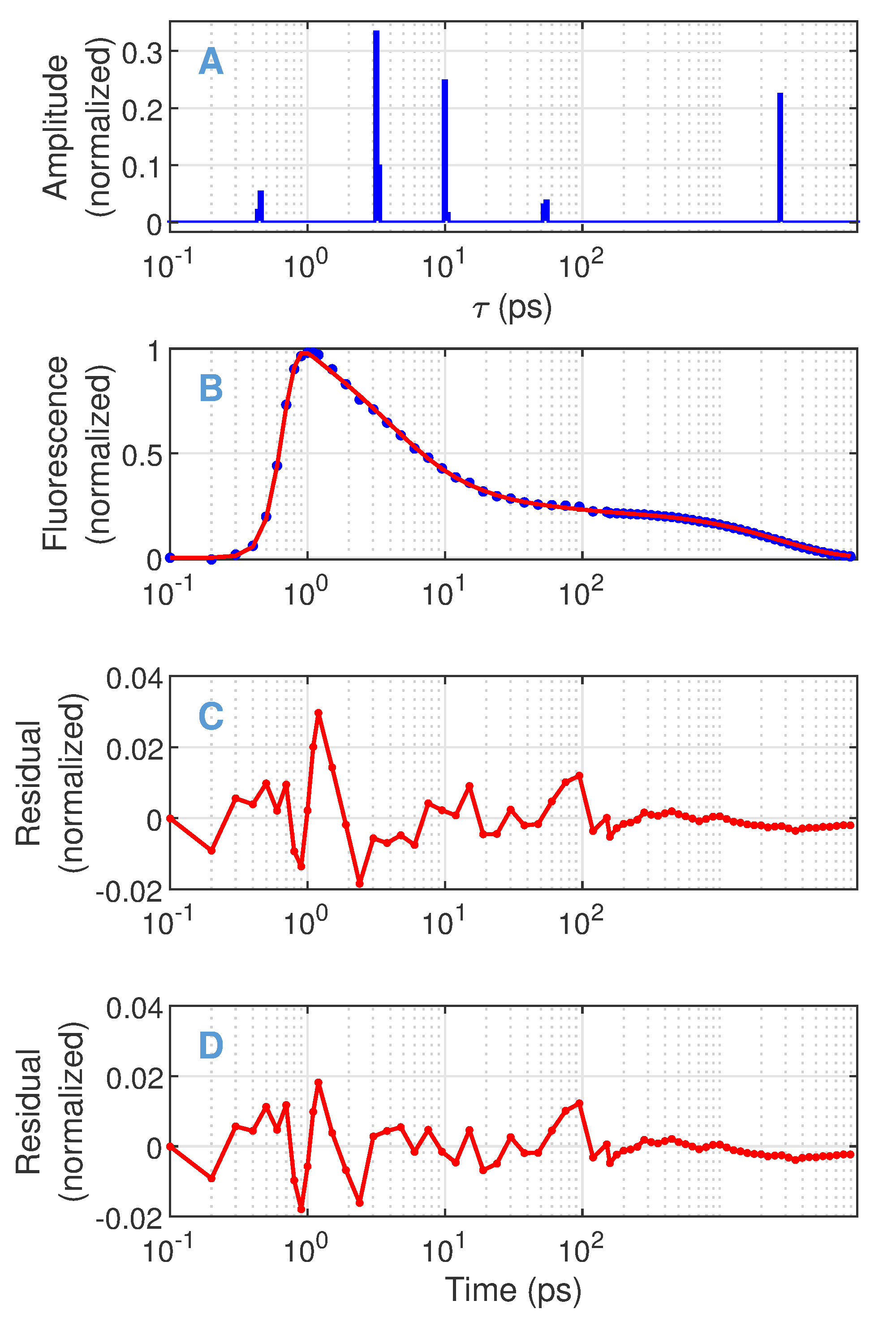

Supplement: S6 Fig — Wavelength: 520 nm. (A) Distribution over time constants. (B) Experimental kinetics (blue) and fit (red) by the distribution presented in (A). (C) Residual of the fit presented in (B). (D) Residual of the correcting exponential fit. (TIF) [file pone.0255675.s006.tif]

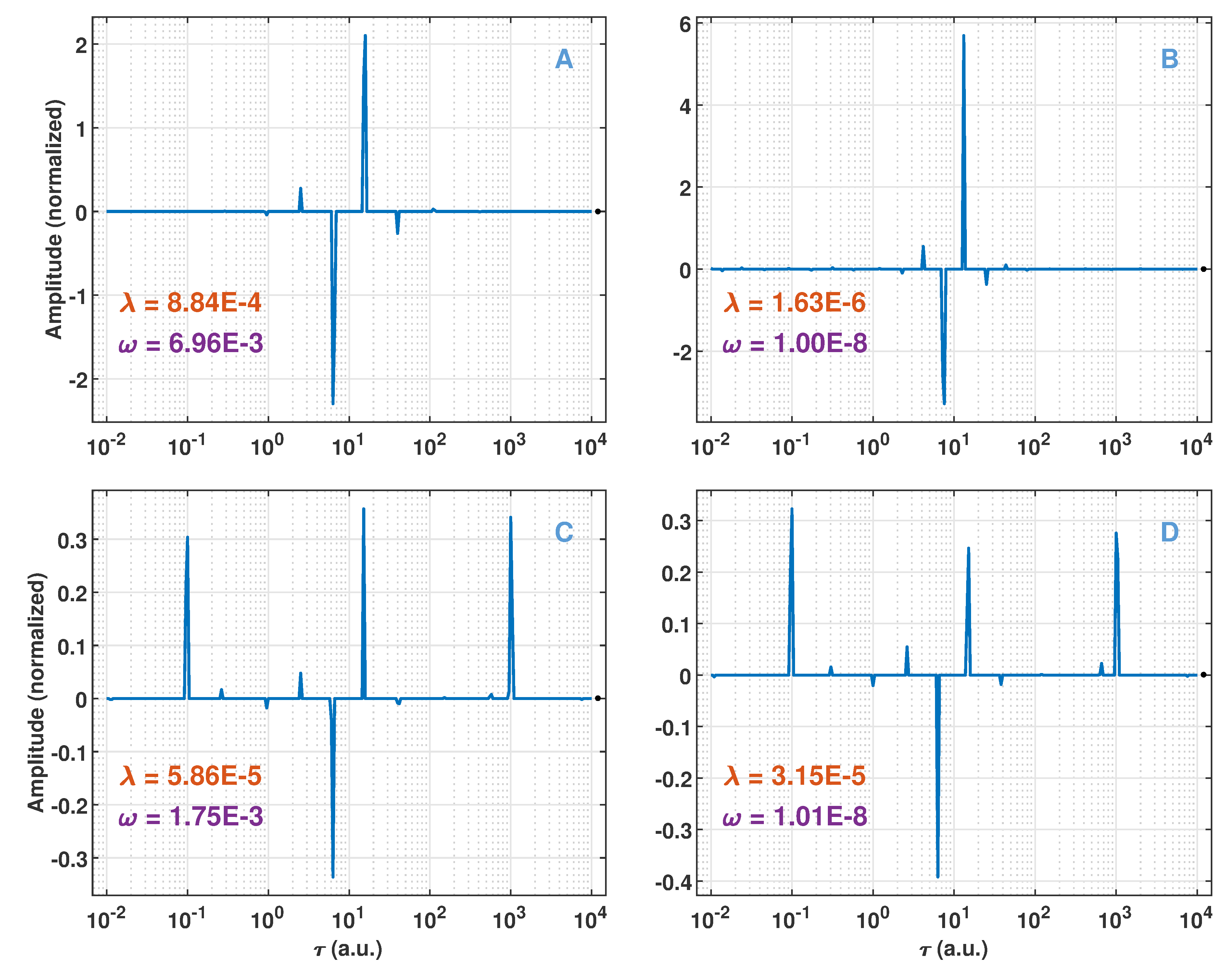

Supplement: S7 Fig — Noise level: σrel = 10−3. For details see the caption of Fig 12 of the main text. (TIF) [file pone.0255675.s007.tif]

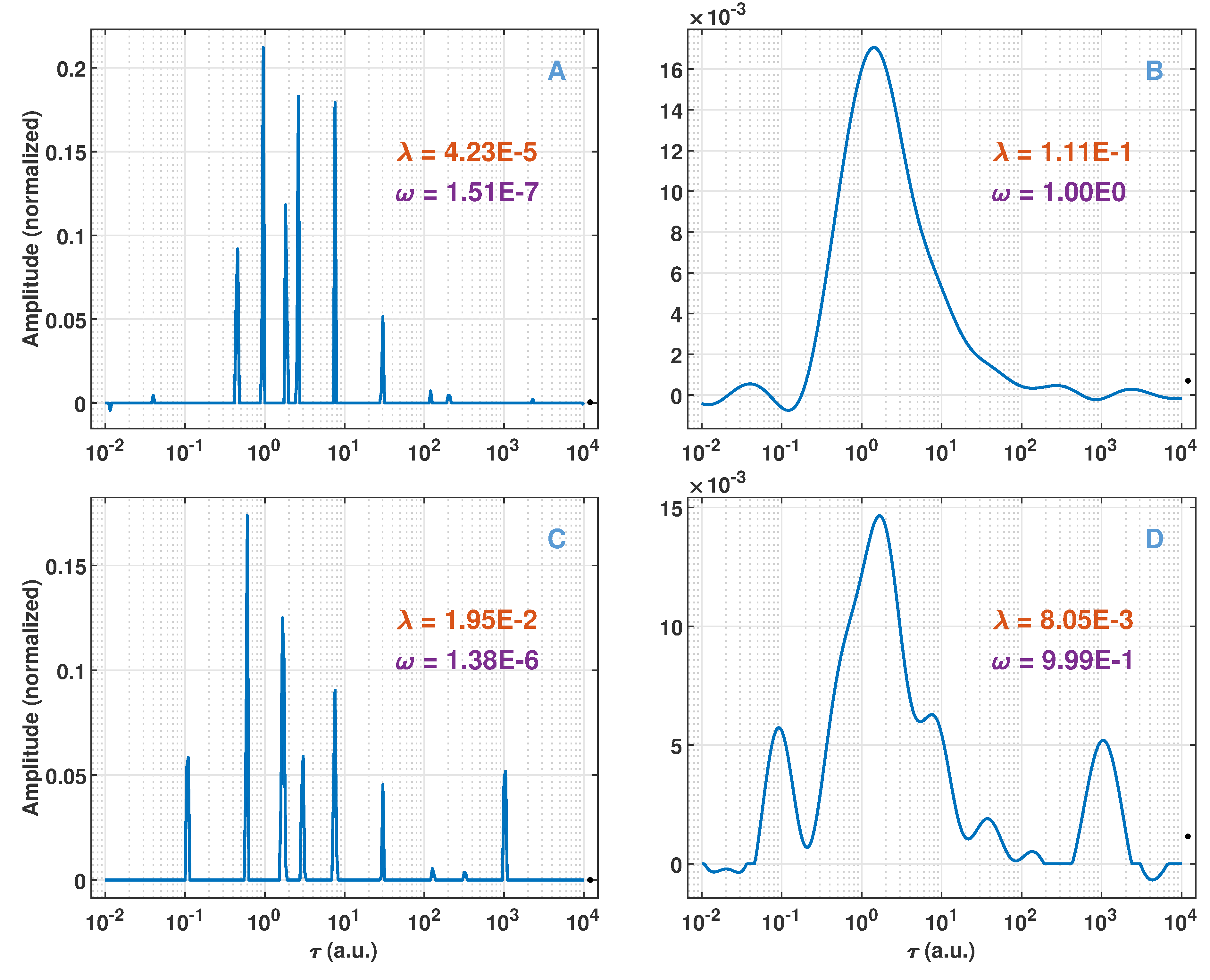

Supplement: S8 Fig — Noise level: σrel = 10−3. For details see the caption of Fig 13 of the main text. (TIF) [file pone.0255675.s008.tif]
