# Supplementary material for: Machine-learning model selection and parameter estimation from kinetic data of complex first-order reaction systems
Source: PLoS One. 2021 Aug 9;16(8):e0255675. doi: 10.1371/journal.pone.0255675 (PMC8352076; doi:10.1371/journal.pone.0255675)
Supplement: S1 Table — (PDF) [file pone.0255675.s009.pdf]

S1 Table. Matrix of first-order microscopic rate constants corresponding to the bR photocycle model presented in Scheme 1 of the main text.

	K1	K2	L1	L2	L3	M1	M2	M3	N1	N2	O	BR
**K1**	-2.00E+06		1.58E+05									
**K2**	1.00E+06	-5.01E+05		2.00E+05								
**L1**	1.00E+06		-4.75E+05									
**L2**		5.01E+05	3.16E+05	-4.51E+05		2.51E+06						
**L3**					-3.98E+06		2.00E+06					
**M1**				2.51E+05		-2.71E+06	1.58E+04					
**M2**					3.98E+06	2.00E+05	-2.02E+06	3.98E+02				
**M3**							1.00E+04	-1.40E+03	1.26E+03			
**N1**								1.00E+03	-2.26E+03	1.26E+02		
**N2**									1.00E+03	-5.24E+02	2.51E+03	
**O**										3.98E+02	-3.51E+03	
**BR**											1.00E+03	
