# Supplementary material for: Machine-learning model selection and parameter estimation from kinetic data of complex first-order reaction systems
Source: PLoS One. 2021 Aug 9;16(8):e0255675. doi: 10.1371/journal.pone.0255675 (PMC8352076; doi:10.1371/journal.pone.0255675)
Supplement: S2 Table — The prediction was calculated by Algorithm 1 on the simulated data at different noise levels (σrel). λ and ω are the selected hyperparameters, MSE refers to the mean square error of the fit. (PDF) [file pone.0255675.s010.pdf]

S2 Table. The predicted time constants (τ) and amplitudes (A) obtained at different noise levels, compared to the true values. The prediction was calculated by Algorithm 1 on the simulated data at different noise levels (σ_{rel}). λ and ω are the selected hyperparameters, MSE refers to the mean square error of the fit.

**true values**			**σ_{rel} = 1.E-7**		**σ_{rel} = 1.E-6**		**σ_{rel} = 1.E-5**		**σ_{rel} = 1.E-4**		**σ_{rel} = 1.E-3**		**σ_{rel} = 1.E-2**	
		λ	7.7E-07		2.1E-05		8.1E-03		6.4E-03		6.2E-03		1.1E-01	
		ω	1.4E-04		1.9E-07		1.4E-04		1.4E-04		1.4E-05		3.5E-05	
		MSE	1.146E-10		4.892E-09		1.182E-06		9.194E-07		1.497E-06		9.388E-05	
**τ (s)**	**A***		**τ (s)**	**A**	**τ (s)**	**A**	**τ (s)**	**A**	**τ (s)**	**A**	**τ (s)**	**A**	**τ (s)**	**A**
1.67E-07	3.E-05		1.94E-07	0.003	1.31E-07	0.002								
3.37E-07	0.028		3.29E-07	0.020	3.08E-07	0.021								
**4.77E-07**	**0.107**		**5.19E-07**	**0.095**	**5.59E-07**	**0.101**	**6.31E-07**	**0.155**	**6.23E-07**	**0.153**	**6.23E-07**	**0.153**		
**1.46E-06**	**0.359**		**1.48E-06**	**0.357**	**1.49E-06**	**0.352**	**1.58E-06**	**0.318**	**1.58E-06**	**0.321**	**1.57E-06**	**0.324**	**1.13E-06**	**0.398**
2.65E-06	0.026		2.77E-06	0.022	3.07E-06	0.019					3.98E-06	0.001	**3.33E-06**	**0.053**
**3.90E-05**	**0.164**		**3.90E-05**	**0.157**	**3.75E-05**	**0.160**	**2.95E-05**	**0.118**	**3.00E-05**	**0.120**	**3.02E-05**	**0.120**	**2.22E-05**	**0.076**
**2.61E-04****	**1.045**		**2.34E-04**	**0.737**	**2.25E-04**	**0.643**	**1.81E-04**	**0.505**	**1.84E-04**	**0.507**	**1.85E-04**	**0.507**	**1.29E-04**	**0.521**
**3.74E-04**	**0.632**		**4.68E-04**	**0.325**	**5.35E-04**	**0.238**	**1.17E-03**	**0.091**	**1.13E-03**	**0.099**	**1.15E-03**	**0.104**		
**2.36E-03**	**0.462**		**2.35E-03**	**0.468**	**2.42E-03**	**0.451**	**2.43E-03**	**0.451**	**2.41E-03**	**0.444**	**2.41E-03**	**0.435**	**3.13E-03**	**0.564**
**1.30E-02**	**0.464**		**1.30E-02**	**0.463**	**1.32E-02**	**0.455**	**1.33E-02**	**0.451**	**1.32E-02**	**0.455**	**1.32E-02**	**0.457**	**1.51E-02**	**0.358**
Inf	1.E-15		Inf	3.E-05	Inf	5.E-04					Inf	4.E-04	Inf	0.001
			**False positive components**											
			7.94E-08	0.001										
			1.44E-05	0.001	1.20E-05	0.003								
			5.08E-05	0.018										
			**1.16E-04**	**0.040**	1.00E-04	0.017	**1.01E-04**	**0.037**	**1.03E-04**	**0.038**	**1.00E-04**	**0.044**		
			9.55E-04	0.034	1.17E-03	0.016								
			3.31E-03	0.002	7.24E-03	0.005								

* the amplitude is defined as the maximum absolute value of the corresponding DADS

** unresolved components of 2.58E-04 s and 2.63E-04 s

components in bold refer to those kept in discretization
