# Supplementary material for: Machine-learning model selection and parameter estimation from kinetic data of complex first-order reaction systems
Source: PLoS One. 2021 Aug 9;16(8):e0255675. doi: 10.1371/journal.pone.0255675 (PMC8352076; doi:10.1371/journal.pone.0255675)
Supplement: S3 Table — For details see the legend of S2 Table. (PDF) [file pone.0255675.s011.pdf]

S3 Table. The predicted time constants (τ) and amplitudes (A) obtained with realistic, wavelength- and time-dependent noise compared to the true values. For details see the legend of S2 Table.

true values			real noise	
		λ	3.7E-02	
		ω	4.2E-05	
		MSE	1.612E-05	
**τ (s)**	**A***		**τ (s)**	**A**
1.67E-07	3.E-05			
3.37E-07	0.028			
**4.77E-07**	**0.107**		**7.61E-07**	**0.196**
**1.46E-06**	**0.359**		**1.68E-06**	**0.269**
2.65E-06	0.026			
**3.90E-05**	**0.164**		**2.63E-05**	**0.106**
**2.61E-04****	**1.045**		**1.55E-04**	**0.520**
**3.74E-04**	**0.632**			
**2.36E-03**	**0.462**		**2.59E-03**	**0.538**
**1.30E-02**	**0.464**		**1.42E-02**	**0.412**
Inf	1.E-15			

* the amplitude is defined as the maximum absolute value of the corresponding DADS

** unresolved components of 2.58E-04 s and 2.63E-04 s

components in bold refer to those kept in discretization
