# Supplementary material for: Machine-learning model selection and parameter estimation from kinetic data of complex first-order reaction systems
Source: PLoS One. 2021 Aug 9;16(8):e0255675. doi: 10.1371/journal.pone.0255675 (PMC8352076; doi:10.1371/journal.pone.0255675)
Supplement: Attachment — Submitted filename: PONE-D-21-05497-review.pdf [file pone.0255675.s012.pdf]

Machine learning-based model selection and parameter estimation from kinetic data of complex first-order reaction systems

László Zimányi, Áron Sipos, Ferenc Sarlós,
Rita Nagypál, and Géza I. Groma

PLOS ONE manuscript PONE-D-21-05497
Received by reviewer: February 22, 2021
Date of review: April 2, 2021

Problem and mathematical method

In chemometrics, first-order kinetics studies evidently lead to data-fitting problems. The authors are deeply familiar with the need for analyzing complex systems of chemical reactions. Their contribution is to combine the statistical analysis methods known as group-lasso and elastic net to fit a model of the form

$$\min_X \frac{1}{2}\sum_{k=1}^{p} \|A_k x_{*,k} - b_k\|_2^2 + \lambda \left[\frac{1}{2}(1-\alpha)\sum_{k=1}^{p}\|x_{*,k}\|_2^2 + \alpha \sum_{j=1}^{n} \|x_{j,*}\|_2\right],$$

where $x_{j,*}$ and $x_{*,k}$ are the jth row and kth column of an unknown $n \times p$ matrix X, and there are data matrices A_k and vectors b_k for each k. This new model is called the group elastic net problem (GENP). The challenge is to find suitable parameters λ and α that balance fits $A_k x_{*,k} \approx b_k$ against the solution norms $\|x_{*,k}\|_2$ and the number of rows $x_{j,*}$ that are exactly zero.

Known methods of cross-validation (CV) and Bayesian optimization (BO) are employed for purposes that I cannot confidently summarize. Without judging the effectiveness of the GENP and the use of CV and BO, I hope that this review helps clarify what the authors wish to say. Sometimes it helps to omit a few words ("less is more"). Sometimes the authors' clarification is needed.

Questions and suggestions are on the scanned manuscript (with most figures omitted). The main ones follow.

Details

1. (Title) "Machine-learning-based model selection ..." needs the extra hyphen, but really it should be "Machine-learning model selection ..." as in the abstract line 20: "a machine-learning algorithm ...".

2. (Abstract, line 21) A newly derived algorithm is being summarized. "derived" seems more suitable than "applied".

3. (Abstract, line 26) "corresponding software" is of more interest when described as "available software".

4. (Introduction, line 64) Clarify "temperature, pH, build models".

5. (p5 and later) Displayed problems of the form "minimize $[\phi(x)]$" can be written "minimize $\phi(x)$" without brackets.

6. (p7 line 138) Clarify "the one global fit". Is "global fit" an established method? "Global multiexponential fitting" is used in the abstract.

7. (p8 line 162) Problem (6) would need to vary both λ and α to deliver a spectrum of dense-to-sparse solutions.

8. (p9 line 170) The future tense never seems essential or appropriate in a paper or book. The paper and book exist already. A reader is happy to learn what is there, not what will be there.

9. (p10 line 202) Take care with the definition of "support".

10. (p12 line 236 and elsewhere) The rule is that only one of ", which" or "that" is correct. Knuth calls "which" without comma a "wicked which".

11. (p17 line 359) Clarify "the L_2 penalty term in (7)". There are two L_2 terms in (7).

12. (p21 line 442) Clarify "The 3-steps BO is needed, because BO is time consuming, hence the 2D version ...". Do you mean "The 3-step BO is needed because BO is time-consuming and the 2D version ...".

13. (p23) Table 1's caption mentions Algorithm 1 and Algorithm 2 before Algorithm 2 has been given. Can you mention Algorithm 2 on p22?

14. (p24 line 493) We should not run a sentence into an Algorithm or Theorem or Lemma.

15. (p25 lines 522–524) Clarify "As seen in Fig 6, steps 1–3 of Algorithm 1 here also select a low value for ω, ensuring a minimal support size and a high value of λ corresponding to 5 features". Why "here also"? The algorithm steps may lead to a high λ and small support, but "ensuring" doesn't seem the right word.

16. (p31 line 631) Clarify "light-induce process".

17. (References) The titles of journal papers don't need capitals. The journal names do (and they are often in italic). Book titles and dissertation titles also need caps.

18. (pp 38–60) Figures and Tables are significantly more useful if their captions summarize what the figures and tables show. Readers should not have to guess.
