# Supplementary material for: Machine-learning model selection and parameter estimation from kinetic data of complex first-order reaction systems
Source: PLoS One. 2021 Aug 9;16(8):e0255675. doi: 10.1371/journal.pone.0255675 (PMC8352076; doi:10.1371/journal.pone.0255675)
Supplement: Attachment — Submitted filename: PONE-D-21-05497-scanned.pdf [file pone.0255675.s013.pdf]

Received 22 Feb 2021
Reviewed 02 Apr 2021

PLOS ONE

# Machine learning-based model selection and parameter estimation from kinetic data of complex first-order reaction systems

--Manuscript Draft--

| | |
|---|---|
| **Manuscript Number:** | PONE-D-21-05497 |
| **Article Type:** | Research Article |
| **Full Title:** | Machine learning-based model selection and parameter estimation from kinetic data of complex first-order reaction systems |
| **Short Title:** | Machine learning-based analysis of complex first-order reaction kinetics |
| **Corresponding Author:** | Géza I. Groma, Ph.D.<br>Biological Research Centre<br>Szeged, HUNGARY |
| **Keywords:** | applied mathematics; chemometrics; physical sciences; physical chemistry; biophysics; biophysical simulation; computational biology; theoretical biology; reaction kinetics; protein kinetics; time-resolved spectroscopy; first-order reactions; exponential fitting; fitting experimental data; analysis of kinetic data; complex systems; machine learning; statistical learning; algorithm; statistical inference; hypothesis testing; sparse modeling; sparse parameters; dense solution; sparse solution; lasso; group lasso; group-lasso; hyperparameters; elastic net; cross-validation; Bayesian optimization; bacteriorhodopsin; bacteriorhodopsin photocycle; FAD; fluorescence kinetics; FAD fluorescence kinetics; ultrafast fluorescence kinetics; fit spectroscopic data |
| **Abstract:** | Dealing with a system of first-order reactions is a recurrent problem in chemometrics, especially in the analysis of data obtained by spectroscopic methods applied on complex biological systems. Here we argue that global multiexponential fitting, the still common way to solve this kind of problems has serious weaknesses, in contrast to the available contemporary methods of sparse modeling. Combining the advantages of group-lasso and elastic net – the statistical methods proven to be very powerful in other areas – we obtained an optimization problem tunable to result in from very sparse to very dense distribution over a large pre-defined grid of time constants, fitting both simulated and experimental multiwavelength spectroscopic data with very high performance. Moreover, it was found that the optimal values of the tuning hyperparameters can be selected by a machine-learning algorithm based on a Bayesian optimization procedure, utilizing a widely used and a novel version of cross-validation. The applied algorithm recovered very exactly the true sparse kinetic parameters of an extremely complex simulated model of the bacteriorhodopsin photocycle, as well as the wide peak of hypothetical distributed kinetics in the presence of different levels of noise. It also performed well in the analysis of the ultrafast experimental fluorescence kinetics data detected on the coenzyme FAD in a very wide logarithmic time window. We conclude that primary application of the presented algorithms – implemented in the corresponding software – covers the wide area of studies on light-induced physical, chemical and biological processes to be carried out by different spectroscopic methods. Especially, a high demand for this kind of analysis is expected from the emerging ultrafast multidimensional infrared and electronic spectroscopic techniques providing very large and complex datasets. In addition, simulations based on our methods could also help in designing technical parameters of future experiments for the verification of a particular hypothetical model. |
| **Order of Authors:** | László Zimányi |
| | Áron Sipos |
| | Ferenc Sarlós |
| | Rita Nagypál |
| | Géza I. Groma, Ph.D. |
| **Additional Information:** | |

Powered by Editorial Manager® and ProduXion Manager® from Aries Systems Corporation

| Question | Response |
|---|---|
| **Financial Disclosure**<br><br>Enter a financial disclosure statement that describes the sources of funding for the work included in this submission. Review the submission guidelines for detailed requirements. View published research articles from *PLOS ONE* for specific examples.<br><br>This statement is required for submission and **will appear in the published article** if the submission is accepted. Please make sure it is accurate.<br><br>**Unfunded studies**<br>Enter: *The author(s) received no specific funding for this work.*<br><br>**Funded studies**<br>Enter a statement with the following details:<br>• Initials of the authors who received each award<br>• Grant numbers awarded to each author<br>• The full name of each funder<br>• URL of each funder website<br>• Did the sponsors or funders play any role in the study design, data collection and analysis, decision to publish, or preparation of the manuscript?<br>• **NO** - Include this sentence at the end of your statement: *The funders had no role in study design, data collection and analysis, decision to publish, or preparation of the manuscript.*<br>• **YES** - Specify the role(s) played.<br><br>* typeset | LZ, FS, NZ, GG; GINOP-2.3.2-15-2016-00001; Economic Development and Innovation Operative Programme of Hungary; https://www.palyazat.gov.hu/node/56577<br><br>LZ; K-124922; National Research, Development and Innovation Office of Hungary; https://nkfih.gov.hu<br><br>AS; NKFIH PD-121170; National Research, Development and Innovation Office of Hungary; https://nkfih.gov.hu<br><br>LZ, GG; 2018-1.2.1-NKP-2018-00009; National Research, Development and Innovation Office of Hungary; https://nkfih.gov.hu<br><br>The funders had no role in study design, data collection and analysis, decision to publish, or preparation of the manuscript. |
| **Competing Interests**<br><br>Use the instructions below to enter a competing interest statement for this submission. On behalf of all authors, disclose any competing interests that could be perceived to bias this work—acknowledging all financial support and any other relevant financial or non-financial competing interests. | The authors have declared that no competing interests exist. |

*Powered by Editorial Manager® and ProduXion Manager® from Aries Systems Corporation*

| | |
|---|---|
| This statement is required for submission and **will appear in the published article** if the submission is accepted. Please make sure it is accurate and that any funding sources listed in your Funding Information later in the submission form are also declared in your Financial Disclosure statement.<br><br>View published research articles from *PLOS ONE* for specific examples.<br><br>**NO authors have competing interests**<br><br>Enter: *The authors have declared that no competing interests exist.*<br><br>**Authors with competing interests**<br><br>Enter competing interest details beginning with this statement:<br><br>*I have read the journal's policy and the authors of this manuscript have the following competing interests: [insert competing interests here]*<br><br>* typeset | |
| **Ethics Statement**<br><br>Enter an ethics statement for this submission. This statement is required if the study involved:<br><br>• Human participants<br>• Human specimens or tissue<br>• Vertebrate animals or cephalopods<br>• Vertebrate embryos or tissues<br>• Field research<br><br>Write "N/A" if the submission does not require an ethics statement.<br><br>General guidance is provided below. Consult the submission guidelines for detailed instructions. **Make sure that all information entered here is included in the Methods section of the manuscript.** | N/A |

| | |
|---|---|
| **Format for specific study types**<br><br>**Human Subject Research (involving human participants and/or tissue)**<br>• Give the name of the institutional review board or ethics committee that approved the study<br>• Include the approval number and/or a statement indicating approval of this research<br>• Indicate the form of consent obtained (written/oral) or the reason that consent was not obtained (e.g. the data were analyzed anonymously)<br><br>**Animal Research (involving vertebrate animals, embryos or tissues)**<br>• Provide the name of the Institutional Animal Care and Use Committee (IACUC) or other relevant ethics board that reviewed the study protocol, and indicate whether they approved this research or granted a formal waiver of ethical approval<br>• Include an approval number if one was obtained<br>• If the study involved *non-human primates*, add *additional details* about animal welfare and steps taken to ameliorate suffering<br>• If anesthesia, euthanasia, or any kind of animal sacrifice is part of the study, include briefly which substances and/or methods were applied<br><br>**Field Research**<br><br>Include the following details if this study involves the collection of plant, animal, or other materials from a natural setting:<br>• Field permit number<br>• Name of the institution or relevant body that granted permission | |
| **Data Availability**<br><br>Authors are required to make all data underlying the findings described fully available, without restriction, and from the time of publication. PLOS allows rare exceptions to address legal and ethical concerns. See the PLOS Data Policy and FAQ for detailed information. | Yes - all data are fully available without restriction |

| | |
|---|---|
| A Data Availability Statement describing where the data can be found is required at submission. Your answers to this question constitute the Data Availability Statement and **will be published in the article**, if accepted.<br><br>**Important:** Stating 'data available on request from the author' is not sufficient. If your data are only available upon request, select 'No' for the first question and explain your exceptional situation in the text box.<br><br>Do the authors confirm that all data underlying the findings described in their manuscript are fully available without restriction? | |
| **Describe where the data may be found in full sentences. If you are copying our sample text, replace any instances of** XXX **with the appropriate details.**<br><br>• If the data are **held or will be held in a public repository**, include URLs, accession numbers or DOIs. If this information will only be available after acceptance, indicate this by ticking the box below. For example: *All XXX files are available from the XXX database (accession number(s) XXX, XXX.).*<br>• If the data are all contained **within the manuscript and/or Supporting Information files**, enter the following: *All relevant data are within the manuscript and its Supporting Information files.*<br>• If neither of these applies but you are able to provide **details of access elsewhere**, with or without limitations, please do so. For example:<br><br>*Data cannot be shared publicly because of [XXX]. Data are available from the XXX Institutional Data Access / Ethics Committee (contact via XXX) for researchers who meet the criteria for access to confidential data.*<br><br>*The data underlying the results presented in the study are available* | After acceptence all the data as well as the sofware applied in this study will be made available in a public repository (probably GitHub). |

| | |
|---|---|
| *from (include the name of the third party and contact information or URL).*<br>• This text is appropriate if the data are owned by a third party and authors do not have permission to share the data.<br><br>* typeset | |
| Additional data availability information: | Tick here if the URLs/accession numbers/DOIs will be available only after acceptance of the manuscript for publication so that we can ensure their inclusion before publication. |

[illegible] [illegible]

# Machine learning-based model selection and parameter estimation from kinetic data of complex first-order reaction systems

*László Zimányi[1], Áron Sipos[1], Ferenc Sarlós[1], Rita Nagypál[1,2] and Géza I. Groma[1]**

[1]Institute of Biophysics, Biological Research Centre, Eötvös Loránd Research Network, H-6701, Szeged, Hungary

[2]Doctoral School of Physics, University of Szeged, H-6720, Szeged, Hungary

*Corresponding author

E-mail: groma.geza@brc.hu

# Abstract

Dealing with a system of first-order reactions is a recurrent problem in chemometrics, especially in the analysis of data obtained by spectroscopic methods applied on complex biological systems. Here we argue that global multiexponential fitting, the still common way to solve this kind of problems has serious weaknesses, in contrast to the available contemporary methods of sparse modeling. Combining the advantages of group-lasso and elastic net – the statistical methods proven to be very powerful in other areas – we obtained an optimization problem tunable to result in from very sparse to very dense distribution over a large pre-defined grid of time constants, fitting both simulated and experimental multiwavelength spectroscopic data with very high performance. Moreover, it was found that the optimal values of the tuning hyperparameters can be selected by a machine-learning algorithm based on a Bayesian optimization procedure, utilizing a widely used and a novel version of cross-validation. The applied algorithm recovered very exactly the true sparse kinetic parameters of an extremely complex simulated model of the bacteriorhodopsin photocycle, as well as the wide peak of hypothetical distributed kinetics in the presence of different levels of noise. It also performed well in the analysis of the ultrafast experimental fluorescence kinetics data detected on the coenzyme FAD in a very wide logarithmic time window. We conclude that primary application of the presented algorithms – implemented in the corresponding software – covers the wide area of studies on light-induced physical, chemical and biological processes to be carried out by different spectroscopic methods. Especially, a high demand for this kind of analysis is expected from the emerging ultrafast multidimensional infrared and electronic spectroscopic techniques providing very large and complex datasets. In addition, simulations based on our methods could also help in designing technical parameters of future experiments for the verification of a particular hypothetical model.

## Introduction

From classical flash photolysis [1-8] to the recent methods of ultrafast time-resolved spectroscopy [9-14], light-induced kinetic studies – especially those carried out on macromolecules – face the challenge of analyzing a complex scheme of reactions. One reason for such a complexity is related to the lengthy cascade of the reactions initiated by photoexcitation. A typical example of that is the sequence of photointermediates of retinal proteins, including the very complicated scheme of bacteriorhodopsin (bR) [15] photocycle. Another reason of complexity lies in the heterogeneity of the conformational states and/or the microenvironment of a chromophore studied by time-resolved fluorescence [9, 11] or transient absorption [14]. Regardless of the degree of complexity, in most of the above problems it can be supposed that the individual steps of the reaction scheme can be well approximated by first-order kinetics. In such a case the problems can be handled by the standard methods for solving a system of linear homogeneous differential equations of first order [16-18]. Briefly, a given scheme of $n$ reaction components can be characterized by an $n \times n$ microscopic rate matrix $\mathbf{K}$, the non-diagonal $K_{i,j}$ elements of which represent the rate constant of the reaction from component $j$ to component $i$, and $K_{i,i}$ is the negative sums of the outward rates from component $i$. The corresponding macroscopic rate constants and decay-associated spectra (DAS) or difference spectra (DADS) [18] – characterizing the experimentally observable kinetic data – can be calculated by solving the eigenvalue problem of $\mathbf{K}$, with satisfying the initial conditions. Due to the existence of constraints among the elements of the matrix, its eigenvalues are always real [17]. If the eigenvalues are also non-degenerate, as generally supposed in routine

analyses, the solution of the above system of differential equations can be expressed as a sum of exponential terms.

From the viewpoint of the experimentalist, the problem to solve is just the reverse of the scheme outlined above. In most cases the observed data do not hold direct information on the time-dependent concentration of the reaction components. Instead, the data typically originate from a kind of spectroscopic experiments and are represented as a set a kinetics detected at different wavelengths. The most ambitious aim of determining the **K** matrix of a given reaction scheme only from these data, called target analysis [18], is impossible, due to the numerous unknown factors. Such an analysis requires to repeat the experiment under varied parameters, e.g. temperature, pH, build models on how the rate constants depend on these parameters and make assumption on the spectrum of the participating components [6, 19]. An interesting novel approach for handling this problem for a relatively simple set of light-induced reactions utilizes a deep learning network trained with synthetic time-resolved spectra [20]. In lack of the needed high amount of experimental data and a priori knowledge, the common way of the analysis is aimed in solving the apparently much simpler problem of determining the macroscopic rate constants and the corresponding DAS/DADS by global fitting with $n$ exponential terms [21]. Unfortunately, even the solution of this reduced task faces serious problems:

*P1* In many cases there is no well-established prior knowledge available for supposing that all the participating reactions are really of first order.

*P2* In many cases the experimental data hold relatively poor information content since only a relatively low number of data points are available in a wide range of time.

*P3* The number of the components $n$ is not known in advance.

*P4* The nonlinear fit requires pre-estimation of all unknown parameters.

*P5* There is no guarantee for reaching the true global minimum, which is even not necessarily unique.

*P6* Exponential fitting is inherently an ill-posed problem: low error on the input data generates high uncertainty on the estimated parameters [22, 23].

Most of the above problems can be avoided if instead of discrete exponentials the predicted result is characterized by a distribution on a quasi-continuous space of the time constants and the nonlinear regression problem is extended by a regularizing penalty term [11, 22-27]. In a recent paper [28] we argued to solve the problem

$$\text{minimize}\left[\frac{1}{2}\|\mathbf{b}-\mathbf{A}\mathbf{x}\|_2^2+\lambda\|\mathbf{x}\|_1\right] \tag{1}$$

where $\mathbf{b}$ is the vector of the experimental data with length of $m$, the element $b_i$ of which is taken at the time $t_i$, $\mathbf{A}$ is an $m\times n$ matrix – called the design (or measurement) matrix – with elements of

$$A_{ij}=\exp\left(-t_i/\tau_j\right), \tag{2}$$

$\tau_j$ is an element of the vector $\boldsymbol{\tau}$ of length $n$, consisting of a series of pre-defined time constants, $\mathbf{x}(\boldsymbol{\tau})$ is the distribution to be determined, $\lambda$ is a positive hyperparameter and the $L_1$ and (the not squared) $L_2$ norm of a vector $\mathbf{v}$ are defined as

$$\|\mathbf{v}\|_1=\sum_{i=1}^{n}|v_i|, \qquad \|\mathbf{v}\|_2=\sqrt{\sum_{i=1}^{n}v_i^2}\,. \tag{3}$$

In the literature of signal processing the problem defined in Eq (1) is termed Basis Pursuit Denoising (BPDN) [29], while in statistics its widely used name is lasso for Least Absolute Selection and Shrinkage Operator [30]. Here we will use the latter, more current acronym, but maintain the standard notations of signal processing [31] .The most important property of the lasso

is that it not only guarantees a regularized solution $\mathbf{x}$, but also a sparse one [32], in accordance with the principle of parsimony, a fundamental rule in model selection [33]. Sparsity ensures a close connection to the original discrete exponential terms. Regularization and sparsity together minimize the appearance of invalid features in the solution due to noise, hence handle *P6*. Since for a given problem described by Eq (1) a fixed value of $\lambda$ unequivocally determines the number of peaks in the solution, *P3* gets eliminated. In addition, the lasso is a convex – but not certainly strictly convex - problem, reducing the difficulties with *P4* and *P5*. Problem *P2* is partially handled by the possibility of obtaining sparse solution even if $n \gg m$ – the favorable condition to gain detailed information on the distribution $\mathbf{x}$ – without introducing extra information into the solution which is not contained in the data themselves. Recently lasso regularization has been applied for the analysis of time-resolved spectroscopic data in other laboratories, and it is an option in the PyLDM package [34, 35].

The aim of the present study is exceeding the capabilities of the simple lasso method in three main directions. First, making possible to analyze multidimensional kinetic data, taking into account the correlations among them. Such a need is obvious e.g. for spectroscopic data, where one expects that the kinetics at every wavelength can be characterized by the same set of time constants. Here we show that the problem can be solved by an extension of lasso, called group-lasso [31, 36]. Second, targeting the still unresolved problem *P1*, we find that another extension, elastic net [37] with an additional hyperparameter does a very good job in controlling the sparsity of the solution $\mathbf{x}$ continuously from a very low to a very high level. Finally, and most importantly, applying the arsenal of modern statistics [38] – particularly cross-validation [39, 40] and Bayesian optimization [41, 42] – we constructed a machine learning system for the task of completely automatic model selection. This task is equivalent to determining the value of the two

hyperparameters exclusively from the data, corrupted by noise of an unknown level. The excellent performance of the above methods is demonstrated on a simulated dataset based on a rather complex model of the photocycle of bR as well as on experimentally determined ultrafast fluorescence kinetic data taken on the coenzyme flavin adenine dinucleotide (FAD).

# Theoretical basis

In this subsection we briefly outline the statistical methods used in this work. For more details we recommend the excellent introductory books of Hastie and coworkers [32, 38].

## Methods for parameter estimation

### The group-lasso

The group-lasso is an extension of the lasso defined in Eq (1) for the case when some groups of the elements of $\mathbf{x}$ are in correlation. Partitioning $\mathbf{x}$ into subvectors $\mathbf{x} = (\mathbf{x}_1, \ldots, \mathbf{x}_G)$, where the elements of any $\mathbf{x}_g$ form a correlated set of values, the group-lasso problem [36] is defined as

$$\text{minimize}\left[\frac{1}{2}\|\mathbf{b} - \mathbf{A}\mathbf{x}\|_2^2 + \lambda \sum_{g=1}^{G} \|\mathbf{x}_g\|_2\right]. \tag{4}$$

This definition ensures that for any $g$ either all or none of the elements of $\mathbf{x}_g$ will be nonzero [32]. This property of the solution is like the one global fit provides for discrete exponential terms. Obviously if each subvector $\mathbf{x}_g$ is of length one, Eq (4) is equivalent to Eq (1).

In the special case when the kinetic data are obtained by a kind of spectroscopic methods one expects a correlation among the elements of $\mathbf{x}$ corresponding to different wavelengths but the same value of the time constant. On the other hand, no such a correlation is expected among the

elements corresponding to different time constants. In this case the above partitioning of $\mathbf{x}$ and the building of a giant design matrix related to that is rather inconvenient, a more natural representation is a matrix form. Supposing that the observations were taken at $p$ wavelengths defined in a vector $\mathbf{w} = (w_1, \ldots, w_p)$, the unknown distribution can be arranged in an $n \times p$ matrix $\mathbf{X}$, whose element $x_{jk}$ corresponds to time constant $\tau_j$ and wavelength $w_k$. Let $\mathbf{x}_{j,*}$ and $\mathbf{x}_{*,k}$ denote the $j^{th}$ row and $k^{th}$ column of $\mathbf{X}$, respectively. The kinetic data themselves are arranged in a set of vectors $\mathbf{b}_k$, $k = 1,.., p$, corresponding to wavelength $w_k$. The design matrix $\mathbf{A}_k$ is defined individually for each value of $k$. This freedom is useful if the elements of the matrix cannot have the simple form as defined in Eq (2), typically for taking into account the convolution of the signal with the instrument response function of a measuring apparatus [14, 18]. For grouping across the individual elements of each row of matrix $\mathbf{X}$ one can set the group-lasso problem as

$$\text{minimize}\left[\frac{1}{2}\sum_{k=1}^{p}\left\|(\mathbf{b}_k - \mathbf{A}_k\mathbf{x}_{*,k})\right\|_2^2 + \lambda\sum_{j=1}^{n}\left\|\mathbf{x}_{j,*}\right\|_2\right]. \tag{5}$$

Obviously, Eq (5) can be extended with other parameters, like additional spaces of wavelength in the case of multidimensional vibrational or electronic spectroscopy.

**The elastic net**

The elastic-net problem [37] combines an $L_1$ and an $L_2$ penalty in the form of

$$\text{minimize}\left\{\frac{1}{2}\|\mathbf{b} - \mathbf{A}\mathbf{x}\|_2^2 + \lambda\left[\frac{1}{2}(1-\alpha)\|\mathbf{x}\|_2^2 + \alpha\|\mathbf{x}\|_1\right]\right\}, \tag{6}$$

where $\alpha \in [0,1]$ is a second hyperparameter. Since the $L_2$ penalty alone does not induce sparsity in the solution, by variation of $\alpha$ in its whole range results in solutions varying from very dense

to very sparse. One can expect that this property of the elastic net can handle problem *P1*. In addition, for $\alpha < 1$ the elastic-net problem is strictly convex, eliminating problems *P4* and *P5*. Regularization with both $L_1$ and $L_2$ penalties was applied for the analysis of low-resolution NMR relaxation kinetic data [43, 44].

In order to utilize the advantages of both group-lasso and elastic net in this study we will use their combination in the form of

$$\text{minimize}\left\{\frac{1}{2}\sum_{k=1}^{p}\left\|(\mathbf{b}_k-\mathbf{A}_k\mathbf{x}_{\bullet,k})\right\|_2^2+\lambda\left[\frac{1}{2}(1-\alpha)\sum_{k=1}^{p}\left\|\mathbf{x}_{\bullet,k}\right\|_2^2+\alpha\sum_{j=1}^{n}\left\|\mathbf{x}_{j,\bullet}\right\|_2\right]\right\}. \quad (7)$$

In the sequel this optimization problem will be referred as group elastic net problem (GENP). Since the variation of $\alpha$ causes sudden changes in the objective function of Eq (7) if its value approaches the value 1, technically it is more appropriate to calculate with the variable $\omega = 1-\alpha$ in a logarithmic scale, as we will do it in this study.

Solving the GENP with a given set of kinetic data at fixed values of $\lambda$ and $\omega$ provides an estimation for the values of $\mathbf{X}$. To execute this task, one needs a procedure of model selection to determine the value of these hyperparameters.

## Methods for model selection

### Cross-validation

Despite its introduction in the 1970s [39, 40], to our best knowledge the method of cross-validation (CV) has not been applied in the kind of analysis outlined in the main section of the Introduction. For our purposes CV must be considered in the context of a machine learning procedure for model selection [38], which needs independent data for the training and testing phases. A CV procedure solves this task by randomly dividing the same dataset into training and testing data. The most

common CV algorithm is the $k$-fold CV which randomly sorts the whole dataset into $k$ subsets. During machine learning $k$-1 sets are applied for learning, that is, for estimating some model parameters by fitting to these data. Then the remaining single set is applied for testing. In the testing phase the data are compared to the values simulated by the parameters calculated in the learning phase and the mean square error (MSE) is calculated. The procedure is executed $k$ times selecting all subsets once for testing. A certain model is characterized by the mean of the MSE values obtained in each turn. In this way CV is a promising tool for model selection. Models with more free parameters typically result in better goodness of fit than those with fewer ones. However, this effect can be partially due to overfitting of noisy data, leading to a superfluous complexity of the model. One can expect that having a set of models characterized by the different values of one or more hyperparameters, the model selected at the minimum value of mean MSE calculated by CV will have an optimal tradeoff between goodness of fit and complexity. Despite the popularity of $k$-fold CV in model selection, the theoretical justification of this expectation for models applying lasso for parameter estimation is unclear [45]. Moreover, practical simulation results indicate that for these types of models with high-dimensional setting ($n \gg m$) $k$-fold CV tends to bias towards unjustified complexity [46].

In a recent paper [47] Feng and Yu point out that one possible reason of the above bias in $k$-fold CV is that across its different splits the support of the solution $\mathbf{x}$, defined as the subset of its indices holding nonzero values, can change. Averaging the MSEs over these misaligned structures makes unjustified the use of this method for model selection. To deal with this problem the authors suggest a special version of leave-$n_v$-out CV, selecting repeatedly and randomly $n_c$ data from the whole dataset of length $n$ for learning and leaving out $n_v = n - n_c$ for testing [48]. The key point of their algorithm is that in the first step the support of $\mathbf{x}$ is determined by a

penalized estimator like lasso, and the CV is calculated with a restricted design matrix, leaving out its columns with indices not found in the support. In this restricted space no penalty is used, only the maximum likelihood estimator (the first term in Eq (1)) is applied. It is proven that under proper conditions this restricted leave-$n_v$-out CV (RCV($n_v$)) is consistent in hyperparameter selection, in the meaning that with $n \to \infty$ the probability of the selected model being the optimal one approaches the value 1. Obviously, this eliminates the bias problem of $k$-fold CV, as also justified by simulation results.

**Bayesian optimization**

The model selection method described above requires determining the minimum of a black-box function $f(\lambda, \omega)$, the values of which can be calculated by executing a CV procedure at fixed pairs of the hyperparameters $\lambda$ and $\omega$. For lasso-like problems it is common to perform these calculations on a pre-defined path of $\lambda$ values. The main advantage of this method is that a very effective algorithm for solving these problems – implemented e.g. in the popular glmnet toolbox [49] – is based on a pathwise iteration [50]. However, in special cases different, more time-consuming algorithms are needed when the number of the points in the space of hyperparameters has to be kept low. In addition, in this space there is no guarantee for a single minimum, and the local ones could be narrow. Hence, instead of a pre-defined grid an adaptive algorithm for exploring the details around the minima would be advantageous, taking also into account the stochastic nature of MSEs obtained from a CV.

An excellent novel procedure recommended for hyperparameter selection is Bayesian optimization (BO) [41, 42]. In a nutshell, in this method the function to be optimized is modelled as a sample from a Gaussian process, characterized by a proper kernel (or covariance) function

ensuring smoothness. According to the machinery of Bayesian inference [51], the values of $f(\lambda,\omega)$ at the set of the known points define a prior probability based on the Gaussian process for the value at any other point. This information is utilized by an acquisition function for determining the position of the next point at which $f(\lambda,\omega)$ is to be calculated for maximal improvement on the knowledge about the position of its global minimum. Evaluation of $f(\lambda,\omega)$ at the new point results in a posterior probability which in turn is used to update the prior for selection of the next point. As a result, this algorithm adaptively explores the areas around the potential minima much extensively than it does in other regions.

# Methods

## Simulation of the absorption kinetics data from a model of the bR photocycle

Numerous photocycle schemes have appeared in the literature in the past several decades for both the wild type and various mutant bRs based on kinetic visible absorption spectroscopy. Single and parallel schemes differ in that in parallel schemes it is assumed that the sample is heterogeneous with different bR species having different photocycles [52], whereas single schemes assume a homogeneous sample [6]. Even single cycles can be branching [3] or non-branching. The complex kinetics of the intermediates have ruled out a single, unidirectional photocycle and the reversibility of most of the molecular steps is now generally accepted [6, 53, 54]. Various strategies to find the appropriate photocycle and the corresponding microscopic rate constants have been proposed and applied by various investigators [4, 7, 55].

In this study, a complex photocycle scheme was considered to generate the simulated data (Scheme 1), which contains mechanistically necessary steps for the proton pumping function of bR, identified by experiments. This scheme is the result of a synthesis of previously published,

linear, reversible schemes with certain additions. The **K** matrix built from the microscopic rate constants of the transitions is listed in S1 Table. The ultrafast transitions involving the "hot" I and J intermediates were not taken into account, since the published multichannel absorption kinetic data generally start in the 10-100 ns range or later, when these intermediates have already decayed to the K intermediate(s) [10]. Two early, sequential L intermediates, L1 and L2, kinetically and spectrally identified as separate intermediates, were considered according to previous work [56]. Relaxation of L1 to L2 is accompanied by the partial reorientation of the Schiff base NH bond, as revealed by X-ray structural data [57]. Since it is generally observed that K persists much longer than the decay of L1, and L1 was shown to completely decay to L2, the model also included a second, spectrally identical K intermediate in equilibrium with L2. A rationale would be the existence of an initial "hot" K, which decays to L1, and both forms can relax to K2 and L2, respectively, probably by energy dissipation into the protein environment of the chromophore therefore these latter steps were considered unidirectional.

It is assumed that the proton transfer from the Schiff base to the anionic D85 (M1 intermediate) is followed by the reorientation „switch", resulting in the change of access of the retinal Schiff base from the extracellular to the cytoplasmic side (M2). The model allows proton transfer between D85 and the Schiff base even after the „switch"; we included in the scheme therefore an L3 intermediate, spectrally not resolved from L2, as a cul-de-sac from M2. The pathway of the proton in this reaction does not need to retrace the original proton transfer from the Schiff base to D85, and it is assumed that the equilibrium shifts towards Schiff base deprotonation (i.e. M2). The model corresponds to neutral or alkaline pH, where proton release from the extracellular water molecule cluster with pKa = 5.8 takes place [58] as a transition between M substates, i.e. no branching to a low pH path with late proton release was modelled. The protonation equilibrium between the

Schiff base and D85, i.e. the equilibrium between L and M, is expected to shift completely in favour of Schiff base deprotonation after extracellular proton release, due to the mutual effect of the protonation state of D85 and the proton release cluster on their respective proton affinities [59]. The two sequential N states appear by the reprotonation of the Schiff base by D96 and the proton uptake from the cytoplasmic side to D96. These substates have indeed been experimentally separated at high pH by polarized spectroscopy [60] or in mutants [61]. The substates of M and the substates of N were modelled with a single M and N spectrum, respectively. After N2 the recovery of the initial resting state was considered in the model through a single O intermediate. Experimental evidence shows that at the conclusion of the photocycle M, N and O are difficult to separate by visible spectroscopy, a circumstance complicated also by the recovery of the resting state, so this is a realistic trade-off. Based on infrared spectroscopy, the reprotonation of D96 from the cytoplasmic bulk has been reported to take place in two steps [8]. Nevertheless, the reported splitting of the cytoplasmic bulk to D96 proton transfer into two separate steps was not considered in the model. The transfer from N2 to the O intermediate corresponds to retinal reisomerization. The final, unidirectional transition to the resting state combines internal proton transfer from D85 to the extracellular proton release cluster and reversal of any conformational alterations present in the O state [5].

Realistic intermediate spectra extracted from experiment were used in the simulation. The spectra obtained earlier by singular value decomposition with self-modeling, cf. Fig 4 in [56] and Fig S2 in its ~~corresponding~~ Supporting Information, were fitted by the analytical nomogram function for visual pigment spectra [62] to obtain noise-free intermediate spectra (S1 Fig A). The difference spectra of the intermediates were calculated by subtracting the absolute spectrum of the bR resting state from them (S1 Fig B). The kinetics of the individual intermediates (S1 Fig C) were

calculated by solving the eigenvalue problem of the $\mathbf{K}$ matrix as discussed in the Introduction, supposing that at $t = 0$ all intermediate concentrations are zero except for K1. The observable absorption change at time $t$ and wavelength $w$ was calculated as

$$\Delta A(t,w) = \sum_{i=1}^{11} s_i(w) c_i(t) = \sum_{i=1}^{11} D_i(w) e^{-\frac{t}{\tau_i}}, \qquad (8)$$

where $s_i(w)$ and $c_i(t)$ are the difference spectrum and the concentration of the *i*-th intermediate, respectively, $\tau_i$ are the macroscopic time constants (S2 Table left column) and $D_i$ are the corresponding DADS. (Formally an additional component of infinite time constant and zero DADS takes place in the right side of Eq (8) related to the existence of the bR resting state.) $t$ was sampled in 50 points as 9 logarithmically equidistant points per decade in the domain from 100 ns to 43 ms. $w$ was sampled in 38 points in the range of 355-730 nm.

## Ultrafast fluorescence kinetics measurements on FAD

For comparability with the results presented in our previous study [28] the experimental conditions were kept identical to those described therein. The fluorescence kinetics experiments were carried out on samples of 1.5 mM aqueous solution of FAD disodium salt hydrate (Sigma-Aldrich) in 10 mM HEPES buffer at pH = 7.0. A home-made measuring apparatus combined the techniques of fluorescence up-conversion and time-correlated single photon counting (TCSPC) for detection of fast and slow components, respectively. The sample was excited at 400 nm with 150 fs pulses of 80 MHz repetition rate. The fluorescence kinetics were detected at magic angle at 11 wavelengths in the range of 490-590 nm. The up-conversion technique sampled the kinetics in a linear section of 0.1-1.2 ps with a dwell time of 0.1 ps, followed by logarithmically equidistant section up to 300 ps with a logarithmic dwell time – defined as $\log_{10}(t_{i+1}/1ps) - \log_{10}(t_i/1ps)$ – of 0.1. The

TCSPC technique sampled in the range of 0 – 6.38 ns with dwell time of 4 ps, the obtained data were then compressed by averaging into a logarithmically equidistant scale with a logarithmic dwell time of 0.05. The two datasets were merged by fitting a small overlapping section at around 150 ps, resulting in a final one consisting of 69 points and ranging from 0.1 ps to 8.91 ns.

## Simulation of distributed kinetics data

This simulation was based on hypothetical reaction kinetics following the Arrhenius equation

$$k = Ae^{-\frac{E}{RT}}. \tag{9}$$

In arbitrary units Eq (9) can be expressed as

$$k(E) = e^{-\frac{E}{50}}, \tag{10}$$

where the activation energy $E$ was sampled in the interval $[0,400]$ with increment 1 (S2 Fig A red line). It was supposed that the molecular population cannot be characterized by a discrete value of the activation energy but – due to the existence of an assembly of substates – it can be described by a Gaussian distribution $g(E)$ with mean of 200 and $\sigma = 35$ (S2 Fig A blue line). The simulated true solution i.e. the distribution of $g(E)$ over $\tau = 1/k(E)$ is presented in S2 Fig B.

## Implementation of the machine learning procedure

An object-oriented MATLAB toolbox FOkin (First-Order kinetics) was developed for handling all the simulation, parameter estimation and model selection problems in a common software environment. The detailed description of the toolbox is included in its documentation, here we outline the main procedures applied therein.

A fundamental task in our simulation is solving the group-lasso problem ($\omega = 1 - \alpha = 0$) or the GENP ($0 < \omega \leq 1$) defined in Eq (7). We tested the following algorithms for this purpose:

- the blockwise descent algorithm [63] implemented in the glmnet in a MATLAB package [49]
- the simultaneous signal decomposition formulation based on block-coordinate descent implemented in the SPAMS toolbox [64]
- the fast iterative shrinkage-thresholding algorithm (FISTA) [65] implemented in the SPAMS toolbox
- the alternating direction method of multipliers (ADMM) [31] algorithm implemented in a collection of MATLAB functions [66].

Surprisingly, the first three algorithms, performing very well in other problems, showed slow convergence, and/or optimized with poor sparsity on our design matrix. On the other hand, the *group_lasso* function [66] implementing the ADMM algorithm provided excellent sparsity with reasonable convergence rate. The convergence rate was further improved by incorporating simple criterions for adaptive updating of the augmented Lagrangian penalty parameter [31]. The augmented Lagrangian technique applied in ADMM also offered a trivial way for the inclusion of the $L_2$ penalty term in Eq (7). With these modifications the function was incorporated into the FOkin toolbox and all GENPs in this study were solved by that. These calculations were carried out with a time constant vector $\boldsymbol{\tau}$ of 50 logarithmically equidistant points in a decade. If the solution of a GENP was sparse it was discretized by characterizing every contiguous region of nonzero values by a time constant and an amplitude, independently at all wavelengths. The time constant was determined by averaging the elements of the $\boldsymbol{\tau}$ vector falling into the region, applying

the absolute value of the corresponding amplitudes as weighting factors. The amplitude was calculated by adding that of the contributing individual elements.

If the dataset was taken at a single wavelength the group-lasso penalty in Eq (7) turns into the simple lasso formula. In this case the minimization problem can be solved by the Primal-Dual interior method for Convex Objectives (PDCO) [29], implemented in the PDCO MATLAB function [67]. (An earlier version of PDCO is incorporated in the SparseLab toolbox [68] utilized in our previous study [28].) Since PDCO outperforms ADMM in runtime, this algorithm was also incorporated into FOkin, and in this study it was applied for the analysis of distributed kinetics.

Both the *k*-fold CV and the RCV($n_v$) algorithms were implemented in FOkin from scratch, applying parallel calculation on the available CPU cores. The random sorting of the data points into the training and testing groups was carried out independently at every wavelength. In *k*-fold CV the value of *k* was 10, as mostly used in the literature. In RCV($n_v$) the fraction $n_c / n$ was 0.9 and the MSE was calculated by averaging the results of $10^4$ CVs in the restricted space.

The machine learning procedure was based on the automatic hyperparameter selection by BO using the *bayesopt* function of the Statistics and Machine Learning Toolbox of MATLAB which applies the ARD Matérn 5/2 kernel [69]. The optimal value of both $\lambda$ and $\omega$ were searched on a logarithmic scale. For covering a high dynamic range, the objective function was also transformed to be logarithmic. On the joint space of $\lambda$ and $\omega$ the search was carried out on 400 points, if any of the hyperparameters was fixed the number of the points was 100. The *expected-improvement-plus* type of the acquisition function was applied to minimize the chance of missing the true minimum.

For the analysis of the experimental fluorescence kinetics data the temporal instrument response function of the measuring device was described by a Gaussian with mean of $t_0$ and standard

deviation of $\sigma$. Accordingly, the pure exponential terms in the columns of the design matrix Eq (2) were substituted for the analytical function of their convolution with a Gaussian [18]. It was supposed that $\sigma$ is wavelength independent and the wavelength dependence of $t_0$ – related mainly to light dispersion – was modelled by a cubic spline of three knot points of fixed x coordinates. The y coordinates of the knots as well as the value of $\sigma$ were considered as free parameters to be determined from the experimental data. To that end we set a GENP with the data, a reasonable value of $\lambda$, $\omega = 0$ and a wavelength-dependent series of the design matrices with these parameters and applied again the method of BO to determine their optimal values. For the main machine learning procedure the obtained values were kept fixed.

### Software availability

The FOkin toolbox developed for this study is publicly available here: *(to be specified on acceptance)*.

## Results and discussion

### Recovering the macroscopic kinetic parameters of a bR photocycle model from simulated data

The data simulated from the bR photocycle model in Scheme 1 hold several challenges for the recovery of the true macroscopic rate constants and DADSs by a machine learning procedure. First, the amplitude corresponding to three of the true time constants (S2 Table left block in normal face) is less than 3% of the maximal one. (A fourth, very small component with infinite time constant is a calculation error, since it should be of zero amplitude as explained in the description

of Eq (8).) The DADSs of remaining dominating components and the corresponding time constants are presented in Fig 1A and 1B, respectively. The second challenge is that the time constant of the two largest DADSs ($2.63\times10^{-4}$s and $2.58\times10^{-4}$s) are obviously unresolvable. In addition, the DADS of these components (dotted lines) show definite mirror symmetry in the high wavelength region, and even their sum (dashed line) remains the largest spectrum. The kinetic data generated from the true parameters are presented in Fig 1C and 1D in temporal and spectral representation, respectively.

The 10-fold CV curve calculated from the above data with relative noise of $10^{-3}$ is presented in Fig 2A. The blue dots represent the points sampled by BO based on GENP with $\omega = 1$, determining a model mean (points predicted on a dense grid after the sampling process, red curve) which has a well-defined minimum in the space of $\lambda$. In contrast to that, the MSE calculated from the solution of GENP without CV on a grid of $\lambda$ is obviously monotonic (cyan curve). As expected with $\omega$ = 1, and in harmony with our previous results [28], the solution corresponding to the minimum is sparse (Fig 2B) it consists of narrow peaks, referred to as features in the following. Also, as a consequence of the grouping penalty, the position of the features is equal at all wavelengths. However, as also expected, the 10-fold CV is biased towards the more complex models [46], manifested in the high number of features and especially in the false doublets at $\sim 2\times10^{-3}$s and $\sim 1\times10^{-2}$. As a treatment for this problem, the model selection procedure was repeated with RCV($n_v$). As seen in Fig 2C in this case the value of the selected $\lambda$ is higher by more than an order of magnitude than that preferred by 10-fold CV. Accordingly, the selected model is much simpler (Fig 2D). This finding excellently agrees with the theoretical results of Feng and Yu, as well as with their calculations with simulated and real data [47].

Utilizing the superior performance of RCV($n_v$) we set up the following machine learning algorithm to be applied:

---

**Algorithm 1**

---

1. Execute a 2D BO optimization on a wide range of both $\lambda$ and $\omega$, applying RCV($n_v$) with the GENP.
2. Fix the optimal value of $\lambda$ obtained in step 1 and execute a BO on $\omega$ only.
3. Fix the optimal value of $\omega$ obtained in step 2 and execute a BO on $\lambda$ only.
4. With the optimal value of $\omega$ and $\lambda$ obtained in step 2 and step 3, respectively, solve the GENP.
5. If the solution in step 4 is sparse, discretize that.

---

The 3-steps BO is needed, because BO is time consuming, hence the 2D version with a reasonable number of iterations yields only a rough estimation of the hyperparameters. For simplicity we refer these steps as model selection and steps 4 and 5 as parameter estimation. Note however, that the algorithm can be also considered as a hierarchical model selection setting. On one level the hyperparameters are selected by BO, while on the other one the nonzero elements are selected from the solution of the GENP.

Algorithm 1 was used to analyze the simulated data with relative noise ranging from $10^{-7}$ to $10^{-2}$. The detailed results are summarized in S2 Table, here we compare the characteristics obtained with a small ($10^{-7}$) and a large ($10^{-3}$) value of $\sigma_{rel}$. The results of the 3-steps model selection are presented in Fig 3, also indicating the model errors and noise errors of BO. Since $\lambda$ controls mainly the number of features, while $\omega$ the width of them, and in this way the support size averaged over the wavelength space, these parameters are also shown (purple curves). It is clearly observable that the algorithm automatically handles the presence of noise in an optimal way. A

higher noise level would cause unjustified complexity in the solution of the GENP but the selected hyperparameters – much higher $\lambda$ and somewhat lower $\omega$ – effectively compensate this unwanted tendency. In addition, the selected value of $\omega$ in both cases is very low, falling into the range where the average support size is around its minimum, ensuring perfect sparsity.

The results of steps 4 and 5 of Algorithm 1 are shown in Table 1 and Fig 4A and 4B, comparing them with the true values. For a compact presentation, the amplitudes in Table 1 represent the maximum absolute value of the corresponding DADSs shown in Fig 4B. According to the table at $\sigma_{rel} = 10^{-7}$ all the 10 finite and resolvable true components are recovered by the algorithm. In addition, it resulted in 6 false positive features with low amplitudes. Neglecting the components having amplitude less than 5% of the largest one, 7 true and 1 false positive of them remain (bold in the table and plotted in Fig 4B). In this selection the remaining false positive is the lowest one (black in Fig 4B). As indicated by red arrows in Fig 4A, the position of all valid features is very close to that of their true counterparts. At $\sigma_{rel} = 10^{-3}$ 7 finite and resolvable true components are recovered with 1 false positive one. The above neglecting affects 1 true feature. In this case the position of the recovered features is less perfect, a considerable shift is observable for the one at $3.74 \times 10^{-4}$ s.

**Table 1. Kinetic parameters (τ and A) predicted at low and high noise levels on the simulated data by Algorithm 1 and Algorithm 2 (8 exponentials).** MSE refers to the mean square error of the fit.

| true values | | $\sigma_{rel}$ = 1.E-7 | | | | $\sigma_{rel}$ = 1.E-3 | | | |
|---|---|---|---|---|---|---|---|---|---|
| | | Algorithm 1 | | Algorithm 2 | | Algorithm 1 | | Algorithm 2 | |
| | | MSE = 1.146E-10 | | MSE = 1.134E-08* | | MSE = 1.497E-06 | | MSE = 6.744E-07 | |
| τ (s) | A | τ (s) | A | τ (s) | A | τ (s) | A | τ (s) | A |
| 1.67E-07 | 3.E-05 | 1.94E-07 | 0.003 | | | | | | |
| 3.37E-07 | 0.028 | 3.29E-07 | 0.020 | | | | | | |
| **4.77E-07** | **0.107** | **5.19E-07** | **0.095** | **4.97E-07** | **0.115** | **6.23E-07** | **0.153** | **6.23E-07** | **0.160** |
| **1.46E-06** | **0.359** | **1.48E-06** | **0.357** | **1.53E-06** | **0.367** | **1.57E-06** | **0.324** | **1.62E-06** | **0.322** |
| 2.65E-06 | 0.026 | 2.77E-06 | 0.022 | **1.99E-06** | **0.065** | 3.98E-06 | 0.001 | | |
| **3.90E-05** | **0.164** | **3.90E-05** | **0.157** | **3.92E-05** | **0.163** | **3.02E-05** | **0.120** | **3.83E-05** | **0.162** |
| **2.61E-04**** | **1.045** | **2.34E-04** | **0.737** | **2.53E-04** | **0.856** | **1.85E-04** | **0.507** | **2.56E-04** | **0.839** |
| **3.74E-04** | **0.632** | **4.68E-04** | **0.325** | **4.07E-04** | **0.451** | **1.15E-03** | **0.104** | **4.15E-04** | **0.448** |
| **2.36E-03** | **0.462** | **2.35E-03** | **0.468** | **2.38E-03** | **0.458** | **2.41E-03** | **0.435** | **2.38E-03** | **0.454** |
| **1.30E-02** | **0.464** | **1.30E-02** | **0.463** | **1.31E-02** | **0.461** | **1.32E-02** | **0.457** | **1.30E-02** | **0.463** |
| Inf | 1.E-15 | Inf | 3.E-05 | | | Inf | 4.E-04 | | |
| | | **False positive components** | | | | | | | |
| | | 7.94E-08 | 0.001 | | | | | | |
| | | 1.44E-05 | 0.001 | | | | | | |
| | | 5.08E-05 | 0.018 | | | | | | |
| | | **1.16E-04** | **0.040** | | | **1.00E-04** | **0.044** | **1.43E-04** | **0.058** |
| | | 9.55E-04 | 0.034 | | | | | | |
| | | 3.31E-03 | 0.002 | | | | | | |

*MSE of the corrected fit keeping all the 17 components is 6.134E-11

**unresolved components of 2.58E-04 s and 2.63E-04 s

components in bold refer to those kept after discretization

The DADSs derived in step 4 (Fig 4B) perfectly recover the shape of the true ones. However, the amplitude of the two largest spectra (corresponding to the true components of $2.61\times10^{-4}$ s and $3.74\times10^{-4}$ s) is considerably smaller than that of their true counterparts, especially in the case of high noise. Obviously, this anomaly is an inherent drawback of Algorithm 1, based on the concept of fitting with penalties. Namely, by definition both the $L_1$ and $L_2$ penalties have higher controlling effect on the components of higher amplitudes. The only way for correcting this unwanted side effect is to lift the constraint imposed by them and execute a simple exponential fitting, the method we highly argued against in the Introduction. Note, however, that at the present point of the analysis this step is completely justified, on the grounds of information already acquired by Algorithm 1. Indeed, since the solutions are sparse, we have learned that the first-order model is correct. We also know the number of the exponentials, and almost correctly even their parameters. This means that the solution obtained by Algorithm 1 is very close the global minimum of the pure multiexponential fitting problem. Accordingly, we suggest the following extension of the algorithm:

---

**Algorithm 2**

---

1. Execute Algorithm 1.
2. Execute a global multiexponential fitting with the result of discretization obtained in step 1 as starting parameters.

---

The results of Algorithm 2 with the 8 dominating components kept above are presented in Table1, by blue arrays in Fig 4A and in Fig 4C. In the case of $\sigma_{rel}=10^{-7}$ the positions of the components were almost correct already after step 1 and hardly changed in step 2. The positive false component disappeared, while a neglected one was recovered. At $\sigma_{rel}=10^{-3}$ the anomalous

shift from $3.74\times10^{-4}$ is perfectly compensated. In both cases the diminished DADS amplitudes got closer to the true values. Notably, the solution at the two noise levels became very similar. The detailed results at a single wavelength are shown in S3 Fig. Keeping all the 17 components presented in Table 1 for $\sigma_{rel}=10^{-7}$ and executing the exponential fitting with them causes minimal changes in the DADSs of the dominating ones (S4 Fig). Compared to the result of step 1, after step 2 the MSE reduced by a factor of two. Overall, for the dominating components the correction by exponential fitting gets the estimated parameters considerably closer to the true values and reduces the difference in the solution at different noise levels.

In summary, it was found that Algorithm 2 is a very sophisticated method for recovering the macroscopic time constants and DADSs corresponding to a very complicated photocycle model in a wide range of error levels, provided that the absolute value of their amplitudes reaches at least a few percent of the maximal one. Under this low threshold level both positive and negative false components emerge. The selected model of bR photocycle leads to three such minor components, hence their justification from the experimental data seems to be unsolvable even at a very low error level. The analysis of such real experimental data by the methods applied in this study will be published elsewhere.

## Analysis of ultrafast experimental fluorescence kinetic data on FAD

The input dataset obtained by ultrafast fluorescence kinetic measurements is presented in Fig 5. On the strength of the knowledge base gathered on the above simulated data, we applied Algorithm 2 also for this experimental dataset. As seen in Fig 6, steps 1-3 of Algorithm 1 here also select a low value for $\omega$, ensuring a minimal support size and a high value of $\lambda$ corresponding to 5 features. The final results of Algorithm 2 are presented in Table 2 and Fig 7, with details in S5 Fig.

As expected from the hyperparameters, the solution after step 1 of Algorithm 2 is very sparse (Fig 7A). All finite time constants are kept after discretization. Both the time constants and the DADSs (Table 2, Fig 7B) are similar to what was presented in Fig 7 and 8 of our previous study [28] carried out on a dataset obtained from a similar experiment and analyzed by lasso with a manually selected reasonable value of $\lambda$. The main difference is the complete wavelength independence of the time constants due to the group-lasso penalty applied in the present study. For the same reason here the shape of the DADSs is smoother. Step 2 of Algorithm 2 hardly affected the position of the features (arrows in Fig 7A), but considerably increased the DADS corresponding to time constants of 8.4 ps and 0.35 ps. Meanwhile the MSE changed by less than a factor of two (Table 2, S5 Fig C and D). Overall, Algorithm 2 performed similarly on the experimental FAD fluorescence data to how it did on simulated data, corresponding to an entirely different kinetic process modeling the photocycle of bR. The comparison of the MSE values presented in Table 2 and S2 Table indicates – as also seen in S5 Fig – that the noise level on the experimental data is within the range used in the simulations. The drop in the level of the residuals above 100 ps (S5 Fig C and D) is due to change of the measuring technique from fluorescence up-conversion to TCSPC.

**Table 2. Kinetic parameters (τ and A) predicted from the fluorescence kinetic data by Algorithm 1 and Algorithm 2 (5 exponentials).**

| | Algorithm 1 | | Algorithm 2 | |
|---|---|---|---|---|
| λ | 5.5E-02 | | | |
| ω | 1.1E-06 | | | |
| MSE | 5.044E-05 | | 2.978E-05 | |
| | τ (ps) | A | τ (ps) | A |
| | **4.5E-01** | **0.171** | **3.5E-01** | **0.213** |
| | **3.2E+00** | **0.436** | **2.0E+00** | **0.393** |
| | **1.0E+01** | **0.301** | **8.4E+00** | **0.408** |
| | **5.4E+01** | **0.077** | **5.1E+01** | **0.085** |
| | **2.8E+03** | **0.253** | **2.8E+03** | **0.253** |
| | Inf | 0.001 | | |

components in bold refer to those kept in discretization

## Analysis of distributed kinetics

The excellent model selection properties of Algorithm 2 on the above sparse kinetics naturally raise the question: how does it behave if the underlying distribution is not actually sparse? Indeed, conformational heterogeneity in proteins can be manifested in distributed kinetics in their functions like ligand binding [2] or folding [70]. Following such kinetics, the truly exciting question is whether steps 1-3 of Algorithm 1 will force on them a relatively good approximation with a sparse distribution or will be able to automatically adjust the value of $\omega$ high enough resulting in the correct dense solution. To answer this question, we simulated a hypothetical dense distribution over the time constants as described in Methods and depicted in S2 Fig B. As shown in Fig 8 the corresponding kinetic curve is hardly distinguishable from that calculated from a single discrete value at the maximum of the simulated activation energy distribution (S2 Fig A blue line). The analysis of the simulated distributed kinetics was carried out with relative noise levels of $10^{-4}$, $10^{-3}$ and $10^{-2}$.

The results of steps 1-3 of Algorithm 1 with $\sigma_{rel} = 10^{-3}$ are presented in the left column of Fig 9. The algorithm behaved as it did for the truly sparse distributions by selecting a very low value of $\omega$ in the range of the minimal support size. Accordingly, the solution obtained in step 4 consists of 3 features of minimal width in the range of the true dense distribution. The residual of the fit shows an anomalously uneven structure (Fig 10 left column).

As a matter of fact, the above failure of Algorithm 1 is not surprising. Actually, it is based on RCV($n_v$) procedure which was preferred over 10-fold CV just because it selects simpler models. Apparently, our purpose now is to move to the opposite direction towards the more 'complex' solutions. To explore this route the model selection steps were repeated with 10-fold CV instead of RCV($n_v$), the results of which are presented in the right column of Fig 9. According to panel B

this method selected $\omega = 1$, involving all points of the $\boldsymbol{\tau}$ vector into the support. At the same time, the selected $\lambda$ moved down as compared to that selected by RCV($n_v$) (Fig 9 C). The solution of the GENP calculated with these hyperparameters results in a single broad feature, beyond a negligible one at $\tau = \infty$. The corresponding residual is random with the expected error level (Fig 10 right column). The obtained distribution is practically indistinguishable from the true one up to $\sigma_{rel} = 10^{-3}$ and kept reasonably similar even at $\sigma_{rel} = 10^{-2}$ (Fig 11). In contrast to these results on the dense true distribution, retesting the bR and FAD data analyzed above with 10-fold CV still resulted in low values of $\omega$, similarly to RCV($n_v$), hence not jeopardizing the conclusions drawn above.

Note that the dense character of the obtained solutions does not mean that the underlying model is actually more complex than a sparse one, e.g. which is shown in the left panel of Fig 10A. The solution is dense only in the sense that it is represented by a wide support in the space of our pre-selected points of time constants. However, this is not a fortunate representation of a Gaussian (the true distribution) which can be characterized by only three parameters (location, width, amplitude) in the continuous space, hence satisfying the principle of parsimony. In contrast to that the sparse solution after discretization needs six parameters (three locations and three amplitudes) for its characterization.

In summary, the model selection based on 10-fold CV recovered a dense true distribution as correctly as RCV($n_v$) did with a sparse one. However, a dense solution means that our fundamental hypothesis that the true model is sparse failed, hence it is better to look for other types of models that can be characterized by a low number of parameters. On the grounds of the results of this study the suggested final algorithm for the analysis of unknown kinetics hypothesized to be of first order is the following:

**Algorithm 3**

1. Execute a 2D BO optimization on a wide range of both $\lambda$ and $\omega$, applying 10-fold CV with the GENP.
2. Fix the optimal value of $\lambda$ obtained in step 1 and execute a BO on $\omega$ only.
3. If the value of $\omega$ is not low the expected solution is not sparse. Go to step 6.
4. The expected solution is sparse. Execute Algorithm 2.
5. Return.
6. The algorithm reached the limits of its valid range: the kinetics cannot be described by first-order reactions. It is still worth to execute steps 1-4 of Algorithm 1. Depending on the shape of the solution obtained try to analyze the data with different sorts of models.

It is out of the scope of this study to investigate how low value of $\omega$ in step 3 of this algorithm is generally needed to ensure sparsity. As a guideline Fig 3B and Fig 6B indicate that $\omega = 10^{-4}$ is a safe upper limit for that.

# Conclusions

Here we find that group elastic net is a powerful and flexible way for the analysis of kinetic data. With properly selected hyperparameters $\lambda$ and $\omega$ it provides a sparse recovery of kinetic parameters describing a system of first-order reactions, even of a very complex scheme. Other range of the hyperparameters results in dense solution clearly indicating that the reaction is not of first order. We also find that the proper value of $\lambda$ and $\omega$ can be found automatically by a machine learning algorithm, utilizing a combination of the classical *k*-fold cross-validation and the novel

RCV($n_v$) version of that. Bayesian optimization turned out to be an ideal tool for solving the corresponding minimization problem.

Our results can be extended in future studies in many directions. One interesting way is to apply the method to kinetics known not to be of first order. The shape of the dense solutions to be obtained perhaps can be categorized for the utilization of other type of machine learning mechanisms which in turn can help to find the proper model in which the kinetics can be described by a low number of parameters. An interesting special case is a system consisting of both first-order and other types of reactions.

This study applied the sophisticated methods of statistics to build the machine learning algorithms from the point of view of the experimentalist, without care whether the strict theoretical conditions are satisfied for their justified application. For example, in the derivation of RCV($n_v$) Feng and Yu [47] supposed an additively separable penalty while the group-lasso term of GENP we applied does not have this property. Similarly, these authors supposed that the support size of the solution is less than the number of data points, which was clearly broken in our calculations with a Gaussian true distribution. Nonetheless, the applied formulae worked well in practice. For theoretical justification of their application further theoretical studies are needed.

Note that both the simulated and the experimental data applied in this study were obtained from the kinetics of a light-induce process. Indeed, the most complex schemes of first-order reactions can be initiated by ultrashort light pulses on physical, chemical and biological systems. Typically, the initial parts of these schemes take place in electronic and vibrational excited states, frequently followed by relaxation phenomena in the ground-state. Accordingly, we expect that the algorithms presented in this study will be most suitable for the analysis of kinetics acquired by a sort of time-resolved spectroscopy. A special need for them is expected from the emerging ultrafast

multidimensional infrared and electronic spectroscopic techniques [13, 71] providing very large and complex datasets. The ability of our algorithm to automatically recognize nonexponential decays could be utilized in the studies of nonadiabatic systems where the relaxation of the electronically excited state is strongly coupled to coherent nuclear wavepacket motions [72].

In addition to the analysis of experimental data, the presented methods also can serve as tools for designing experiments aimed at proving hypothetical models. Making simulations with different levels of noise like it was presented on bR (S2 Table) one can estimate the value of signal-to-noise ratio required for the demonstration of the existence of non-dominating kinetic components.

# Supporting information

**S1 Table. Matrix of first-order microscopic rate constants corresponding to the bR photocycle model presented in Scheme 1 of the main text.**

**S1 Fig. Spectral and kinetic properties of the components presented in S1 Table.**

**S2 Fig. Construction of kinetic data based on the Arrhenius equation with distributed activation energy.**

**S2 Table. The predicted time constants (τ) and amplitudes (A) compared to the true values.**

**S3 Fig. Detailed results of Algorithm 2 on the simulated data presented in Fig 4 of the main text.**

**S4 Fig. DADS obtained from Algorithm 2 by keeping all the 17 components of the discretization.**

**S5 Fig. Detailed results of Algorithm 2 on the experimental FAD fluorescence kinetic data presented in Fig 7 of the main text.**

Paper titles don't use capitals.
Journals, book titles, thesis titles do.

# References


1. Grossweiner LI. The Study of Labile States of Biological Molecules with Flash Photolysis. In: Augenstein LG, Mason R, Zelle MAX, editors. Adv Radiat Biol. 2: Elsevier; 1966. p. 83-133.
2. Austin RH, Beeson KW, Eisenstein L, Frauenfelder H, Gunsalus IC. Dynamics of Ligand Binding to Myoglobin. Biochemistry. 1975;14(24):5355-73. PubMed PMID: ISI:A1975AX94000021.
3. Váró G, Lanyi JK. Kinetic and spectroscopic evidence for an irreversible step between deprotonation and reprotonation of the Schiff base in the bacteriorhodopsin photocycle. Biochemistry. 1991;30(20):5008-15. doi: 10.1021/bi00234a024.
4. Nagle JF, Zimányi L, Lanyi JK. Testing BR photocycle kinetics. Biophys J. 1995;68(4):1490-9. doi: 10.1016/S0006-3495(95)80321-8.
5. Balashov SP, Lu M, Imasheva ES, Govindjee R, Ebrey TG, Othersen B, et al. The Proton Release Group of Bacteriorhodopsin Controls the Rate of the Final Step of Its Photocycle at Low pH. Biochemistry. 1999;38(7):2026-39. doi: 10.1021/bi981926a.
6. van Stokkum IHM, Lozier RH. Target Analysis of the Bacteriorhodopsin Photocycle Using a Spectrotemporal Model. J Phys Chem B. 2002;106(13):3477-85. doi: 10.1021/jp0127723.
7. Zimányi L. Analysis of the Bacteriorhodopsin Photocycle by Singular Value Decomposition with Self-Modeling: A Critical Evaluation Using Realistic Simulated Data. J Phys Chem B. 2004;108(13):4199-209. doi: 10.1021/jp0364809.
8. Lorenz-Fonfria VA, Saita M, Lazarova T, Schlesinger R, Heberle J. pH-sensitive vibrational probe reveals a cytoplasmic protonated cluster in bacteriorhodopsin. Proc Natl Acad Sci U S A. 2017;114(51):E10909-E18. doi: 10.1073/pnas.1707993114.
9. Pal SK, Peon J, Zewail AH. Biological water at the protein surface: Dynamical solvation probed directly with femtosecond resolution. Proc Natl Acad Sci U S A. 2002;99(4):1763-8. doi: 10.1073/pnas.042697899. PubMed PMID: ISI:000174031100005.
10. Ruhman S, Hou B, Friedman N, Ottolenghi M, Sheves M. Following Evolution of Bacteriorhodopsin in Its Reactive Excited State via Stimulated Emission Pumping. J Am Chem Soc. 2002;124(30):8854-8. doi: 10.1021/ja026426q.
11. Haldar S, Kombrabail M, Krishnamoorthy G, Chattopadhyay A. Monitoring Membrane Protein Conformational Heterogeneity by Fluorescence Lifetime Distribution Analysis Using the Maximum Entropy Method. J Fluoresc. 2010;20(1):407-13. doi: 10.1007/s10895-009-0554-z. PubMed PMID: WOS:000274401600048.
12. van Wilderen LJ, Lincoln CN, van Thor JJ. Modelling Multi-Pulse Population Dynamics from Ultrafast Spectroscopy. PLoS One. 2011;6:e17373. doi: 10.1371/journal.pone.0017373.
13. Zhang Z, Lambrev PH, Wells KL, Garab G, Tan H-S. Direct observation of multistep energy transfer in LHCII with fifth-order 3D electronic spectroscopy. Nature Communications. 2015;6:7914. doi: 10.1038/ncomms8914.
14. Heiner Z, Roland T, Leonard J, Haacke S, Groma GI. Kinetics of Light-Induced Intramolecular Energy Transfer in Different Conformational States of NADH. J Phys Chem B. 2017;120:8037-45. doi: 10.1021/acs.jpcb.7b04753.

15. Lanyi JK. Bacteriorhodopsin. In: Jeon KW, editor. Int Rev Cytol. 187: Academic Press; 1999. p. 161-202.

16. Berberan-Santos MN, Martinho JMG. The integration of kinetic rate equations by matrix methods. J Chem Educ. 1990;67(5):375. doi: 10.1021/ed067p375.

17. Nagle JF. Solving complex photocycle kinetics. Theory and direct method. Biophys J. 1991;59(2):476-87. doi: 10.1016/S0006-3495(91)82241-X.

18. van Stokkum IHM, Larsen DS, van Grondelle R. Global and target analysis of time-resolved spectra. Biochim Biophys Acta. 2004;1657(2-3):82-104. doi: 10.1016/j.bbabio.2004.04.011. PubMed PMID: WOS:000222734200002.

19. Ludmann K, Gergely C, Váró G. Kinetic and Thermodynamic Study of the Bacteriorhodopsin Photocycle over a Wide pH Range. Biophys J. 1998;75(6):3110-9. doi: 10.1016/S0006-3495(98)77752-5.

20. Kollenz P, Herten D-P, Buckup T. Unravelling the Kinetic Model of Photochemical Reactions via Deep Learning. J Phys Chem B. 2020;124(29):6358-68. doi: 10.1021/acs.jpcb.0c04299.

21. Knorr FJ, Harris JM. Resolution of multicomponent fluorescence spectra by an emission wavelength-decay time data matrix. Anal Chem. 1981;53(2):272-6. doi: 10.1021/ac00225a033.

22. Landl G, Langthaler T, Engl HW, Kauffmann HF. Distribution of Event Times in Time-Resolved Fluorescence - the Exponential Series Approach Algorithm, Regularization, Analysis. J Comput Phys. 1991;95(1):1-28. PubMed PMID: ISI:A1991FR89300001.

23. Giurleo JT, Talaga DS. Global fitting without a global model: Regularization based on the continuity of the evolution of parameter distributions. J Chem Phys. 2008;128(11):114114. doi: 10.1063/1.2837293. PubMed PMID: WOS:000254292500017.

24. Livesey AK, Brochon JC. Analyzing the Distribution of Decay Constants in Pulse-Fluorometry Using the Maximum-Entropy Method. Biophys J. 1987;52(5):693-706. PubMed PMID: ISI:A1987K669300003.

25. Siemiarczuk A, Wagner BD, Ware WR. Comparison of the maximum-entropy and exponential series methods for the recovery of distributions of lifetimes from fluorescence lifetime data. J Phys Chem. 1990;94(4):1661-6. doi: 10.1021/j100367a080. PubMed PMID: WOS:A1990CP90300080.

26. Liu YS, Ware WR. Photophysics of Polycyclic Aromatic-Hydrocarbons Adsorbed on Silica-Gel Surfaces .1. Fluorescence Lifetime Distribution Analysis - an Ill-Conditioned Problem. J Phys Chem. 1993;97(22):5980-6. PubMed PMID: ISI:A1993LE95100033.

27. Lorenz-Fonfria VA, Kandori H. Practical aspects of the maximum entropy inversion of the Laplace transform for the quantitative analysis of multi-exponential data. Appl Spectrosc. 2007;61(1):74-84. doi: 10.1366/000370207779701460. PubMed PMID: WOS:000243857100012.

28. Groma GI, Heiner Z, Makai A, Sarlos F. Estimation of kinetic parameters from time-resolved fluorescence data: A compressed sensing approach. RSC Advances. 2012;2(30):11481-90. doi: 10.1039/c2ra21773b. PubMed PMID: WOS:000312145400044.

29. Chen SSB, Donoho DL, Saunders MA. Atomic decomposition by basis pursuit. Siam J SIAM Sci Comput. 1998;20(1):33-61. PubMed PMID: ISI:000075434800003.

30. Tibshirani R. Regression Shrinkage and Selection via the Lasso. Journal of the Royal Statistical Society Series B (Methodological). 1996;58(1):267-88.

31. Boyd S, Parikh N, Chu E, Peleato B, Eckstein J. Distributed Optimization and Statistical Learning via the Alternating Direction Method of Multipliers. Foundations and Trends® in Machine Learning. 2011;3(1):1-122. doi: 10.1561/2200000016.
32. Hastie T, Tibshirani R, Wainwright M. Statistical Learning with Sparsity: The Lasso and Generalizations. Boca Raton, Fl: CRC Press; 2015.
33. Rish I, Grabarnik GY. Sparse Modeling: Theory, Algorithms, and Applications. Herbrich R, Graepel T, editors. Boca Raton, FL: Chapman & Hall/CRC; 2015.
34. Dorlhiac GF, Fare C, van Thor JJ. PyLDM - An open source package for lifetime density analysis of time-resolved spectroscopic data. PLoS Comp Biol. 2017;13(5):e1005528. doi: 10.1371/journal.pcbi.1005528.
35. Smith DA, McKenzie G, Jones AC, Smith TA. Analysis of time-correlated single photon counting data: a comparative evaluation of deterministic and probabilistic approaches. Methods and Applications in Fluorescence. 2017;5(4):042001. doi: 10.1088/2050-6120/aa8055.
36. Yuan M, Lin Y. Model selection and estimation in regression with grouped variables. J Roy Stat Soc Ser B (Stat Method). 2006;68(1):49-67. doi: 10.1111/j.1467-9868.2005.00532.x.
37. Zou H, Hastie T. Regularization and variable selection via the elastic net. J Roy Stat Soc B. 2005;67:301-20. doi: 10.1111/j.1467-9868.2005.00503.x. PubMed PMID: ISI:000227498200007.
38. Hastie T, Tibshirani R, Friedman J. The Elements of Statistical Learning: Data Mining, Inference, and Prediction. 2nd ed: Springer; 2009.
39. Allen DM. The Relationship Between Variable Selection and Data Agumentation and a Method for Prediction. Technometrics. 1974;16(1):125-7. doi: 10.1080/00401706.1974.10489157.
40. Stone M. Cross-Validatory Choice and Assessment of Statistical Predictions. Journal of the Royal Statistical Society: Series B (Methodological). 1974;36(2):111-33. doi: 10.1111/j.2517-6161.1974.tb00994.x.
41. Mockus J. Application of Bayesian approach to numerical methods of global and stochastic optimization. Journal of Global Optimization. 1994;4(4):347-65. doi: 10.1007/BF01099263.
42. Brochu E, Cora VM, de Freitas N. A Tutorial on Bayesian Optimization of Expensive Cost Functions, with Application to Active User Modeling and Hierarchical Reinforcement Learning: arXiv:1012.2599; 2010. Available from: https://arxiv.org/abs/1012.2599.
43. Berman P, Levi O, Parmet Y, Saunders M, Wiesman Z. Laplace inversion of low-resolution NMR relaxometry data using sparse representation methods. Concepts in Magnetic Resonance Part A. 2013;42(3):72-88. doi: 10.1002/cmr.a.21263.
44. Campisi-Pinto S, Levi O, Benson D, Resende MT, Saunders M, Linder C, et al. Simulation-Based Sensitivity Analysis of Regularization Parameters for Robust Reconstruction of Complex Material's T1 − T21H LF-NMR Energy Relaxation Signals. Appl Magn Reson. 2020;51(1):41-58. doi: 10.1007/s00723-019-01173-1.
45. Zhang C-H, Huang J. The sparsity and bias of the Lasso selection in high-dimensional linear regression. Ann Statist. 2008;36(4):1567-94. doi: 10.1214/07-AOS520.
46. Yu Y, Feng Y. Modified Cross-Validation for Penalized High-Dimensional Linear Regression Models. Journal of Computational and Graphical Statistics. 2014;23(4):1009-27. doi: 10.1080/10618600.2013.849200.

47. Feng Y, Yu Y. The restricted consistency property of leave-nv-out cross-validation for high-dimensional variable selection. Statistica Sinica. 2019;29:1607-30. doi: 10.5705/ss.202015.0394.

48. Shao J. Linear Model Selection by Cross-validation. Journal of the American Statistical Association. 1993;88(422):486-94. doi: 10.1080/01621459.1993.10476299.

49. Qian J, Hastie T, Friedlander M, Tibshirani R, Simon N. Glmnet in Matlab 2013. Available from: https://web.stanford.edu/~hastie/glmnet_matlab/.

50. Friedman JH, Hastie T, Tibshirani R. Regularization Paths for Generalized Linear Models via Coordinate Descent. 2010. 2010;33(1):22. Epub 2010-02-02. doi: 10.18637/jss.v033.i01.

51. Gelman A, Carlin JB, Stern HS, Dunson DB, Vehtari A, Rubin DB. Bayesian Data Analysis. third ed. New York: CRC Press; 2013.

52. Hendler RW. An Apparent General Solution for the Kinetic Models of the Bacteriorhodopsin Photocycles. J Phys Chem B. 2005;109(34):16515-28. doi: 10.1021/jp052733h.

53. Váró G, Lanyi JK. Thermodynamics and energy coupling in the bacteriorhodopsin photocycle. Biochemistry. 1991;30(20):5016-22. doi: 10.1021/bi00234a025.

54. Druckmann S, Heyn MP, Lanyi JK, Ottolenghi M, Zimányi L. Thermal equilibration between the M and N intermediates in the photocycle of bacteriorhodopsin. Biophys J. 1993;65(3):1231-4. doi: 10.1016/S0006-3495(93)81166-4.

55. Hendler RW, Shrager RI, Bose S. Theory and Procedures for Finding a Correct Kinetic Model for the Bacteriorhodopsin Photocycle. J Phys Chem B. 2001;105(16):3319-28. doi: 10.1021/jp002362z.

56. Zimányi L, Saltiel J, Brown LS, Lanyi JK. A priori resolution of the intermediate spectra in the bacteriorhodopsin photocycle: the time evolution of the L spectrum revealed. The Journal of Physical Chemistry A. 2006;110(7):2318-21. doi: 10.1021/jp056874v. PubMed PMID: 16480288.

57. Lanyi JK, Schobert B. Mechanism of Proton Transport in Bacteriorhodopsin from Crystallographic Structures of the K, L, M1, M2, and M2′ Intermediates of the Photocycle. J Mol Biol. 2003;328(2):439-50. doi: 10.1016/S0022-2836(03)00263-8.

58. Zimányi L, Váró G, Chang M, Ni B, Needleman R, Lanyi JK. Pathways of proton release in the bacteriorhodopsin photocycle. Biochemistry. 1992;31(36):8535-43. doi: 10.1021/bi00151a022.

59. Balashov SP. Protonation reactions and their coupling in bacteriorhodopsin. Biochim Biophys Acta. 2000;1460(1):75-94. doi: 10.1016/S0005-2728(00)00131-6.

60. Borucki B, Otto H, Heyn MP. Time-Resolved Linear Dichroism and Linear Birefringence of Bacteriorhodopsin at Alkaline pH: Identification of Two N Substates with Different Orientations of the Transition Dipole Moment. J Phys Chem B. 2004;108(6):2076-86. doi: 10.1021/jp035679i.

61. Dioumaev AK, Brown LS, Needleman R, Lanyi JK. Coupling of the Reisomerization of the Retinal, Proton Uptake, and Reprotonation of Asp-96 in the N Photointermediate of Bacteriorhodopsin. Biochemistry. 2001;40(38):11308-17. doi: 10.1021/bi011027d.

62. Stavenga DG, Smits RP, Hoenders BJ. Simple exponential functions describing the absorbance bands of visual pigment spectra. Vision Res. 1993;33(8):1011-7. doi: 10.1016/0042-6989(93)90237-Q.

63. Simon N, Friedman J, Hastie T. A Blockwise Descent Algorithm for Group-penalized Multiresponse and Multinomial Regression: arXiv:1311.6529v1; 2013. Available from: https://arxiv.org/abs/1311.6529.
64. SPAMS toolbox. Available from: http://spams-devel.gforge.inria.fr/index.html.
65. Bach F, Jenatton R, Mairal J, Obozinski G. Optimization with Sparsity-Inducing Penalties. Foundations and Trends® in Machine Learning. 2012;4(1):1-106. doi: 10.1561/2200000015.
66. Boyd S, Parikh N, Chu E, Peleato B, Eckstein J. MATLAB scripts for alternating direction method of multipliers. Available from: https://web.stanford.edu/~boyd/papers/admm/.
67. Saunders M. PDCO: Primal-Dual interior method for Convex Objectives. Available from: http://web.stanford.edu/group/SOL/software/pdco/.
68. SparseLab toolbox. Available from: http://sparselab.stanford.edu.
69. Snoek J, Larochelle H, Adams RP. Practical Bayesian Optimization of Machine Learning Algorithms. In: Jordan MI, LeCun Y, Solla SA, editors. Adv Neural Inf Process Syst. Cambridge, MA, USA: The MIT Press; 2012. p. 2951-9.
70. Kim TW, Lee SJ, Jo J, Kim JG, Ki H, Kim CW, et al. Protein folding from heterogeneous unfolded state revealed by time-resolved X-ray solution scattering. Proc Natl Acad Sci U S A. 2020;117(26):14996-5005. doi: 10.1073/pnas.1913442117.
71. Nuernberger P, Lee KF, Joffre M. Femtosecond Spectroscopy from the Perspective of a Global Multidimensional Response Function. Acc Chem Res. 2009;42(9):1433-41.
72. Stock G, Thoss M. Classical Description of Nonadiabatic Quantum Dynamics. Adv Chem Phys. 2005. p. 243-375.

# Figures

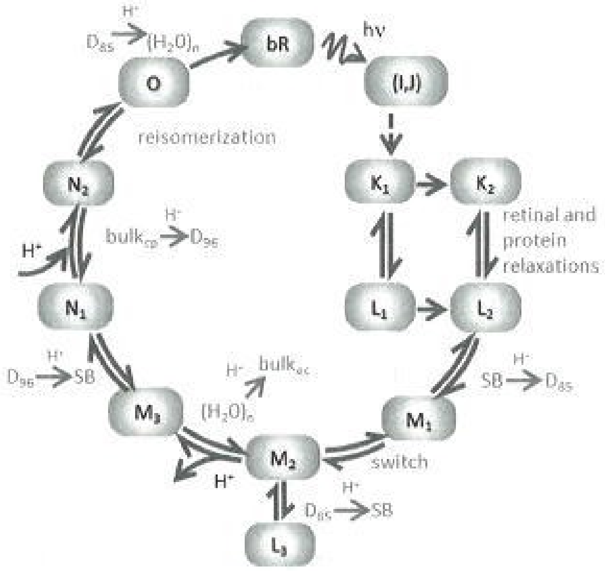


**Scheme 1. Model of the bR photocycle providing input data for the simulations.**

All dark reactions between the intermediates are supposed to be first-order ones. See S1 Table for the corresponding microscopic rate constants.
