# Supplementary material for: Machine-learning model selection and parameter estimation from kinetic data of complex first-order reaction systems
Source: PLoS One. 2021 Aug 9;16(8):e0255675. doi: 10.1371/journal.pone.0255675 (PMC8352076; doi:10.1371/journal.pone.0255675)
Supplement: Attachment — Submitted filename: PONE-D-21-05497R1_review.pdf [file pone.0255675.s015.pdf]

Machine-learning model selection and parameter estimation from kinetic data of complex first-order reaction systems

László Zimányi, Áron Sipos, Ferenc Sarlós,
Rita Nagypál, and Géza I. Groma

PLOS ONE manuscript PONE-D-21-05497R1

Received by reviewer: June 25, 2021
Date of review: July 18, 2021

I thank the authors for attending diligently to the suggested grammatical changes to the (very long) original manuscript. Also for providing a copy of the revised manuscript with important changes highlighted in yellow. I have reviewed these changes and attach a pdf with just a few final suggestions for the yellow sections.

Best wishes to the authors for their ongoing research.
