# Supplementary material for: Machine-learning model selection and parameter estimation from kinetic data of complex first-order reaction systems
Source: PLoS One. 2021 Aug 9;16(8):e0255675. doi: 10.1371/journal.pone.0255675 (PMC8352076; doi:10.1371/journal.pone.0255675)
Supplement: Attachment — Submitted filename: PONE-D-21-05497R1-scanned.pdf [file pone.0255675.s016.pdf]

[illegible]

Received 25 June 2021
Reviewed 18 July 2021

Machine-learning model selection and parameter estimation from kinetic data of complex first-order reaction systems

Commented [GG1]: Extensions and changes other than grammatical and syntactical corrections are highlighted in yellow.

László Zimányi[1], *Áron Sipos*[1], *Ferenc Sarlós*[1], *Rita Nagypál*[1,2] *and Géza I. Groma*[1]*
[1]Institute of Biophysics, Biological Research Centre, Eötvös Loránd Research Network, H-6701, Szeged, Hungary
[2]Doctoral School of Physics, University of Szeged, H-6720, Szeged, Hungary
*Corresponding author
E-mail: groma.geza@brc.hu

Click here to access/download
Supporting Information
S8 Fig.tif

Abstract

Dealing with a system of first-order reactions is a recurrent issue in chemometrics, especially in the analysis of data obtained by spectroscopic methods applied on complex biological systems. We argue that global multiexponential fitting, the still common way to solve such problems, has serious weaknesses compared to contemporary methods of sparse modeling. Combining the advantages of group lasso and elastic net – the statistical methods proven to be very powerful in other areas – we created an optimization problem tunable from very sparse to very dense distribution over a large pre-defined grid of time constants, fitting both simulated and experimental multiwavelength spectroscopic data with high computational efficiency. We found that the optimal values of the tuning hyperparameters can be selected by a machine-learning algorithm based on a Bayesian optimization procedure, utilizing widely used or novel versions of cross-validation. The derived algorithm accurately recovered the true sparse kinetic parameters of an extremely complex simulated model of the bacteriorhodopsin photocycle, as well as the wide peak of hypothetical distributed kinetics in the presence of different noise levels. It also performed well in the analysis of the ultrafast experimental fluorescence kinetics data detected on the coenzyme FAD in a very wide logarithmic time window. We conclude that the primary application of the presented algorithms – implemented in available software – covers a wide area of studies on light-induced physical, chemical, and biological processes carried out with different spectroscopic methods. The demand for this kind of analysis is expected to soar due to the emerging ultrafast multidimensional infrared and electronic spectroscopic techniques that provide very large and complex datasets. In addition, simulations based on our methods could help in designing the technical parameters of future experiments for the verification of particular hypothetical models.

Introduction

From classical flash photolysis [1-8] to recent methods of ultrafast time-resolved spectroscopy [9-14], light-induced kinetic studies – especially those carried out on macromolecules – face the challenge of analyzing a complex scheme of reactions. One reason for such complexity is related to the lengthy cascade of reactions initiated by photoexcitation. A typical example is the sequence of photointermediates of retinal proteins, including the complicated scheme of the bacteriorhodopsin (bR) [15] photocycle. Complexity is also attributed to the heterogeneity of the conformational states and/or the microenvironment of a chromophore studied with time-resolved fluorescence [9, 11] or transient absorption methods [14]. Regardless of the degree of complexity, in most of the above problems we can suppose that the individual steps of the reaction scheme are well approximated by first-order kinetics. In such cases, the problems can be addressed by standard methods designed to solve systems of first-order linear homogeneous differential equations [16-18].

Briefly, the temporal evolution of a given scheme of reaction components can be described by:

$$\frac{d\mathbf{c}(t)}{dt} = \mathbf{K}\mathbf{c}(t), \qquad (1)$$

where $\mathbf{c}(t)$ is an n-vector containing the concentrations of the components at time t and $\mathbf{K}$ is known as the microscopic rate matrix. For $i \neq j$, $K_{i,j}$ represents the rate constant of the reaction from component j to component i, and $K_{i,i}$ is the negative sum of the outward rates from component i. The solution of Eq (1) with appropriate initial conditions requires the solution of the eigenvalue problem of $\mathbf{K}$. Constraints among the elements of the matrix ensure that its eigenvalues are always real [17]. If the eigenvalues are non-degenerate, as generally supposed in

routine analyses, the solution of the differential equations can be expressed as a sum of exponential terms, with rate constants equal to the obtained eigenvalues called macroscopic rate constants. Due to the coupling of the different components in Eq (1), an individual exponential term appearing in its solution – and observable for the experimentalist too – may belong to the time evolution of more than one component. If the data are obtained by a spectroscopy experiment, the spectral distribution of the amplitude of such a term is called decay-associated spectrum (DAS) or difference spectrum (DADS) [18].

In the light of the above possible coupling, because of the numerous unknown factors, it is impossible to achieve the most ambitious aim of determining the $\mathbf{K}$ matrix of a given reaction scheme by target analysis [18], i.e. only from temporal and spectrotemporal experimental data. Such an analysis requires repeating the experiment with various parameters (e.g. temperature and/or pH), building models on how the rate constants depend on these parameters and making assumptions on the spectra of the participating components [6, 19]. An interesting novel approach for handling this problem for a relatively simple set of light-induced reactions utilizes a deep learning network trained with synthetic time-dependent spectra [20]. Without a high amount of experimental data and a priori knowledge, the common approach is to determine the macroscopic rate constants and the corresponding DAS/DADS by global fitting with n exponential terms [21]. Although this is a much simpler task, it still implies problems:

- *P1* In many cases there is no well-established prior knowledge suggesting that all participating reactions are really of first order.
- *P2* Experimental data often provide rather poor information because only a relatively low number of data points are available in a wide time range.
- *P3* The number of components n is not known in advance.

P4 The nonlinear fit requires pre-estimation of all unknown parameters.

P5 There is no guarantee of reaching the true global minimum, which may not be unique.

P6 Exponential fitting is inherently an ill-posed problem: even low error input data generate high uncertainty in the estimated parameters [22, 23].

Most of these issues can be avoided if instead of discrete exponentials the predicted result is characterized by a distribution on a quasi-continuous space of time constants and the nonlinear regression problem is extended by a regularizing penalty term [11, 22-27]. In a recent paper [28] we argued to solve the problem

$$\text{minimize } \frac{1}{2}\|\mathbf{b}-\mathbf{A}\mathbf{x}\|_2^2+\lambda\|\mathbf{x}\|_1 , \tag{2}$$

where $\mathbf{b}$ is the vector of the experimental data with a length of m, the element b_i of which is taken at the time t_i, $\mathbf{A}$ is an $m\times n$ matrix – called the design (or measurement) matrix – with elements

$$A_{ij}=\exp\left(-t_i/\tau_j\right), \tag{3}$$

τ_j is an element of the n-vector $\boldsymbol{\tau}$, consisting of a series of pre-defined time constants, $\mathbf{x}(\boldsymbol{\tau})$ is the distribution to be determined, λ is a positive hyperparameter, and the L_1 and (the not squared) L_2 norm of a vector $\mathbf{v}$ are defined as

$$\|\mathbf{v}\|_1=\sum_{i=1}^{n}|v_i|, \qquad \|\mathbf{v}\|_2=\sqrt{\sum_{i=1}^{n}v_i^2} . \tag{4}$$

In the literature of signal processing, problem (2) is termed Basis Pursuit Denoising (BPDN) [29], while in statistics its widely used name is lasso for Least Absolute Selection and Shrinkage Operator [30]. Here we use the latter, more current acronym, but maintain the standard notations of signal processing [31]. The most important property of the lasso is that it not only guarantees a

regularized solution $\mathbf{x}$, but also a sparse one [32], in accordance with the principle of parsimony, a fundamental rule in model selection [33]. Sparsity ensures a close connection to the original discrete exponential terms. Regularization and sparsity together minimize the appearance of invalid features in the solution due to noise, hence address *P6*. Since for a given problem (2) a fixed value of λ unequivocally determines the number of peaks in the solution, *P3* is eliminated. In addition, the lasso is a convex – but not necessarily strictly convex – problem, which reduces the difficulties with *P4* and *P5*. Problem *P2* is partially handled by the fact that a sparse solution may be obtained even if $n \gg m$ – the favorable condition to gain detailed information on the distribution $\mathbf{x}$ – without introducing information into the solution other than that contained in the data themselves. Recently, lasso regularization has been applied for the analysis of time-resolved spectroscopic data in other laboratories, and it is an option in the PyLDM package [34, 35].

The aim of the present study is to exceed the capabilities of the simple lasso method in three main directions. First, we intend to enable it to analyze multidimensional kinetic data, taking into account the correlations among them. This requirement is obvious e.g. for spectroscopic data, where one expects that the kinetics at every wavelength can be characterized by the same set of time constants. Here we show that the problem can be solved by an extension of the lasso, called group lasso [31, 36]. Second, to target the yet unresolved problem *P1*, we find that another extension, elastic net [37] with an additional hyperparameter, does a very good job in controlling the sparsity of the solution $\mathbf{x}$ continuously from a very low to a very high level. Finally, and most importantly, by applying the arsenal of modern statistics [38] – particularly cross-validation [39, 40] and Bayesian optimization [41, 42] – we have constructed a machine-learning system for completely automatic model selection. This task is equivalent to determining the value of the two hyperparameters exclusively from the data, corrupted by noise of an unknown level. The excellent

performance of the above methods is demonstrated on a simulated dataset based on a rather complex model of the bR photocycle as well as on experimentally determined ultrafast fluorescence kinetic data taken on the coenzyme flavin adenine dinucleotide (FAD). The workflow for the development of the algorithm of the presented methods is depicted in Scheme 1.

Scheme 1. The workflow of building the increasingly complex algorithms for accurate model selection.

Theoretical basis

In this section we briefly outline the statistical methods used in this work. For more details we recommend the excellent introductory books of Hastie and coworkers [32, 38].

Methods for parameter estimation

The group lasso

The group lasso is an extension of the lasso defined in Eq (2) for the case when some groups of the elements of $\mathbf{x}$ are in correlation. Partitioning $\mathbf{x}$ into subvectors $\mathbf{x} = (\mathbf{x}_1, \ldots, \mathbf{x}_G)$, where the elements of any $\mathbf{x}_g$ form a correlated set of values, the group lasso problem [36] is defined as

$$\text{minimize} \frac{1}{2}\|\mathbf{b} - \mathbf{A}\mathbf{x}\|_2^2 + \lambda \sum_{g=1}^{G} \|\mathbf{x}_g\|_2 . \quad (5)$$

This definition ensures that for any g either all or none of the elements of $\mathbf{x}_g$ will be nonzero [32]. This property of the solution is similar to that obtainable by global multiexponential fitting. Obviously, if each subvector $\mathbf{x}_g$ is of length one, Eq (5) is equivalent to Eq (2).

t was sampled at 50 points as 9 logarithmically equidistant points per decade in the domain from 100 ns to 43 ms, while w was sampled at 38 points in the 355-730 nm range.

The effect of random noise in the data on the analysis was tested first by adding uniformly distributed noise of varying amplitude (i.e. standard deviation of its Gaussian distribution relative to the maximal absolute value of the data matrix, σ_{rel}) to the data matrix. Finally, realistic noise spectra were constructed by first filling a matrix of the same size as the data matrix, $\Delta A(t,w)$, with Poisson distributed random numbers of mean zero and realistic amplitude. Then, the spectral and temporal variation of the noise amplitude was modeled in a way consistent with our typical experimental conditions. The noise amplitude appearing in the absorption difference spectra is inversely proportional to the square root of the number of accumulated photons, which depends on the light intensity spectrum, on the gate pulse width of the CCD detector and on the number of accumulated scans contributing to a difference spectrum. Hence, the noise spectra (the columns of the matrix) were first multiplied point by point using the inverse of the square root of a typical light intensity spectrum of a tungsten lamp, measured behind a sample of wild-type bacteriorhodopsin (S2 Fig A). Usual measurements over many decades in time were split into a few time segments with increasing gate pulse width and a varying number of accumulations, to produce a better signal-to-noise ratio where long delay times allow longer integration times. Accordingly, the simulated noise spectra were then divided by the square root of 5, 15 and 40 in the delay segments of [1μs, 20μs], [20μs, 100μs], ≥100μs, respectively, corresponding to typical noise reduction at later delays as compared to the fastest, <1μs delay range (see S2 Fig B). The resulting spectrally and temporally heterogeneous noise matrix was added to the noise-free spectrotemporal data matrix to yield the input data for the analysis with realistic noise.

Ultrafast fluorescence kinetics measurements on FAD

For comparison with the results presented in our previous study [28], the experimental conditions were kept identical to those described therein. The fluorescence kinetics experiments were carried out on samples of 1.5 mM aqueous solution of FAD disodium salt hydrate (Sigma-Aldrich) in 10 mM HEPES buffer at pH = 7.0. A home-made measuring apparatus combined the techniques of fluorescence up-conversion and time-correlated single photon counting (TCSPC) for the detection of fast and slow components, respectively. The sample was excited at 400 nm with 150 fs pulses of 80 MHz repetition rate. The fluorescence kinetics were detected at magic angle conditions at 11 wavelengths in the 490–590 nm range. The up-conversion technique sampled the kinetics in a linear section of 0.1–1.2 ps with a dwell time of 0.1 ps, followed by logarithmically equidistant section up to 300 ps with a logarithmic dwell time – defined as $\log_{10}(t_{i+1}/1ps)-\log_{10}(t_i/1ps)$ – of 0.1. The TCSPC technique sampled in the 0 – 6.38 ns range with a dwell time of 4 ps, the obtained data were then compressed by averaging into a logarithmically equidistant scale with a logarithmic dwell time of 0.05. The two datasets were merged by fitting a small overlapping section at around 150 ps, resulting in a final one consisting of 69 points and ranging from 0.1 ps to 8.91 ns.

Simulation of distributed kinetics data

This simulation was based on hypothetical reaction kinetics following the Arrhenius equation

$$k = Ae^{-\frac{E}{RT}}. \quad (10)$$

In arbitrary units, Eq (10) can be expressed as

Fig 1. Input parameters of the simulation based on Scheme 2.

(A) The dominating DADSs and (B) the corresponding macroscopic time constants. (C) Temporal and (D) spectral representation of the kinetic data calculated from the parameters presented in (A) and (B).

The 10-fold CV curve calculated from the above data with a relative noise of 10^{-3} is presented in Fig 2A. The blue dots represent the points sampled by BO based on GENP with $\omega = 1$, determining a model mean (points predicted on a dense grid after the sampling process, red curve) which has a well-defined minimum in the space of λ. In contrast to that, the MSE calculated from the solution of GENP without CV on a grid of λ is obviously monotonic (cyan curve). As expected with $\omega = 1$, and in accordance with our previous results [28], the solution corresponding to the minimum is sparse (Fig 2B): it consists of narrow peaks, referred to as features in the following. Also, as a consequence of the grouping penalty, the position of the features is equal at all wavelengths. However, as anticipated, the 10-fold CV is biased towards the more complex models [47]. This manifests in the high number of features and especially in the false doublets at $\sim 2\times 10^{-3}$ s and $\sim 1\times 10^{-2}$ s. To eliminate this problem, the model selection procedure was repeated with RCV(n_v). As seen in Fig 2C, in this case the value of the selected λ is by more than an order of magnitude higher than that preferred by 10-fold CV. Accordingly, the selected model is much simpler (Fig 2D). This finding excellently agrees with the theoretical results of Feng and Yu, as well as with their calculations with simulated and real data [48].

Fig 2. Model selection by BO from the simulated data presented in Fig 1C/D at a fixed value of ω = 1.

Noise level: σ_{ref} = 10^{-3}. (A) Mean MSE obtained without (cyan) and with 10-fold CV (blue and red). (C) Mean MSE obtained with RCV(n_v). (B) and (D) Solution of the GENP calculated with the value of λ selected at the minimum presented in (A) and (C), respectively. A line in a particular color in panel (B) and (D) corresponds to a column $\mathbf{x}_{\cdot,\lambda}$ in Eq (8), plotted against the grid of the time constants, while the colors represent different wavelengths. (The black dot beyond the upper limit of τ corresponds to the value of $\tau = \infty$.) The sparsity of the solution is manifested in the low number of features in the form of narrow spikes, representing single exponentials, whose time constant and amplitude are indicated by their location and height, respectively.

I don't see a black dot

Utilizing the superior performance of RCV(n_v) we set up the following machine-learning algorithm.

Algorithm 1

---

1. Execute a 2D BO optimization on a wide range of both λ and ω, applying RCV(n_v) with the GENP.
2. Fix the optimal value of λ obtained in Step 1 and execute a BO on ω only.
3. Fix the optimal value of ω obtained in Step 2 and execute a BO on λ only.
4. With the optimal value of ω and λ obtained in Steps 2 and 3, solve the GENP.
5. If the solution in Step 4 is sparse, discretize it.

The 3-step BO is needed because BO is time-consuming, and the 2D version with a reasonable number of iterations yields only a rough estimation of the hyperparameters. For simplicity, we refer to these steps as model selection and to Steps 4 and 5 as parameter estimation. Note, however, that the algorithm can also be considered as a hierarchical model selection setting. On one level the hyperparameters are selected by BO, while on the other the nonzero elements are selected from the solution of the GENP.

Algorithm 1 was used to analyze the simulated data with relative noise ranging from 10^{-7} to 10^{-2}. The detailed results are summarized in S2 Table, here we compare the characteristics obtained with a small (10^{-7}) and large (10^{-3}) value of σ_{rel}. The results of the 3-step model selection are presented in Fig 3, which also indicates the model and noise errors of BO. Since λ controls mainly the number of features, while ω their width, and consequently the support size averaged over the wavelength space, these parameters are also shown (purple curves). It can be clearly seen that the algorithm automatically handles the presence of noise in an optimal way. A higher noise level would cause unjustified complexity in the solution of the GENP, but the selected hyperparameters – much higher λ and somewhat lower ω – effectively compensate for this unwanted tendency. In addition, the selected value of ω in both cases is very low: it falls into the range where the average support size is around its minimum, ensuring perfect sparsity.

Fig 3. Model selection by BO based on RCV(nv) from the simulated data with different noise levels.

Results of the steps of Algorithm 1. (A) Step 1: BO in the joint space of λ and ω, explored by the BO process at the blue dots. (B) Step 2: BO in the space of ω with the fixed value of λ obtained in Step 1. The purple line (right axis) represents the average support size against

ω. (C) Step 3: BO in the space of λ with the fixed value of ω obtained in Step 2. The purple line (right axis) represents the number of features against λ.

The results of Steps 4 and 5 of Algorithm 1 are shown in Table 1 and Fig 4A and 4B, and are set against the true values. For a compact presentation, the amplitudes in Table 1 represent the maximum absolute value of the corresponding DADSs shown in Fig 4B. According to the table, at $\sigma_{rel} = 10^{-7}$ all the ten finite and resolvable true components are recovered by the algorithm. In addition, the algorithm yielded six false positive features with low amplitudes. Neglecting the components with amplitudes smaller than 5% of the largest one, seven true and one false positive features remain (bold in the table and plotted in Fig 4B). In this selection, the remaining false positive feature is the lowest one (black in Fig 4B). As indicated by red arrows in Fig 4A, the position of all valid features is very close to that of their true counterparts. At $\sigma_{rel} = 10^{-3}$ 7 finite and resolvable true components are recovered with one false positive component. The above neglect affects one true feature. In this case the position of the recovered features is less perfect, and the one at 3.74×10^{-4} s is considerably shifted.

Fig 4. Results of Algorithm 1 and 2 on the selected models presented in Fig 3.

(A) Representation over time constants (Algorithm 1, see captions for Fig 2 B and D for details). The arrows point to the position of the true values (red) and those obtained by Algorithm 2 (valid ones blue, false positive black). (B) Representation by DADSs, neglecting components below 5% of the maximal one (Algorithm 1, dotted) compared to the true DADSs as presented in Figure 1A. (C) DADS obtained by Algorithm 2.

anomalous shift from 3.74×10^{-4} is perfectly compensated. In both cases, the diminished DADS amplitudes become closer to the true values. Notably, the solutions at the two noise levels become very similar. The detailed results at a single wavelength are shown in S4 Fig. Keeping all the 17 components presented in Table 1 under $\sigma_{rel}=10^{-7}$ and executing the exponential fitting with them causes minimal changes in the DADSs of the dominating ones (S5 Fig). Compared to the result of Step 1, after Step 2 the MSE reduced by a factor of two. Overall, for the dominating components, the correction by exponential fitting brings the estimated parameters considerably closer to the true values and reduces the difference in the solution at different noise levels.

Up to this point it was assumed that noise has the property of independent and identical distribution (*iid*) for the whole spectrotemporal dataset. However, this criterion may not be satisfied in real experimental data for two main reasons:

(i) The number of the molecules participating in a chemical reaction network can be very low (e.g., in a single cell). In this case, the occupancies of the species become partially stochastic [71]. This noise-like component clearly depends both on time and the species.

(ii) The experimental procedure introduces noise depending on time and/or wavelength.

For the absorption kinetics of the bR photocycle, the observation of the first effect is completely unrealistic with the present experimental techniques. According to the method of linear noise approximation, the ratio of the classical deterministic and the stochastic part of the kinetics has an order of $\sqrt{N}$, where N is the number of participating molecules [71]. To obtain a reasonable absorption kinetics signal of the bR photocycle, one need to excite $\sim10^{14}$ bR molecules, hence the estimated relative level of noise originating from this intrinsic effect is $\sigma_{rel}=10^{-7}$. The dominating stochastic error in a real absorption kinetics experiment is due to the photon noise of the observation beam. As described in the Methods and shown in S2 Fig, the level of this noise

depends markedly on both time and wavelength. In a real experiment, the average of this noise is usually in the order of $10^{-3} - 10^{-2}$, as compared to the maximal amplitude of the measured signal. The time constants and amplitudes predicted for the data simulated with average noise at $\sigma_{rel} = 3.5*10^{-3}$ are presented in S3 Table. Comparison with S2 Table shows that these parameters fall very well between those corresponding to the cases with *iid* noise of $\sigma_{rel} = 10^{-3}$ and $\sigma_{rel} = 10^{-2}$, proving that the algorithm works perfectly even when the *iid* condition is not satisfied.

In summary, it was found that Algorithm 2 is a very sophisticated method to recover the macroscopic time constants and DADSs corresponding to a very complicated photocycle model in a wide range of error levels, provided that the absolute value of their amplitudes reaches at least a few percent of the maximal amplitude. Under this low threshold level, both positive and negative false components emerge. The selected model of the bR photocycle leads to three such minor components; hence their justification from the experimental data seems to be unsolvable even at a very low noise level. The analysis of such real experimental data by the methods applied in this study will be published elsewhere.

Analysis of ultrafast experimental fluorescence kinetic data on FAD

The input dataset obtained by ultrafast fluorescence kinetic measurements is presented in Fig 5. On the strength of the knowledge base gathered on the above simulated data, we applied Algorithm 2 to this experimental dataset. As seen in Fig 6, Steps 1 to 3 of Algorithm 1 select a low value for ω (leading to a minimal support size), and a high value of λ (corresponding to five features). The final results of Algorithm 2 are presented in Table 2 and Fig 7, with details in S6 Fig. As expected from the hyperparameters, the solution after Step 1 of Algorithm 2 is very sparse (Fig 7A). All finite time constants are kept after discretization. Both the time constants and the

is dense only in the sense that it is represented by a wide support in the space of our pre-selected points of time constants. However, this is not a fortunate representation of a Gaussian (the true distribution) that can be characterized by only three parameters (location, width, amplitude) in the continuous space, hence satisfying the principle of parsimony. In contrast to that, the sparse solution after discretization needs six parameters (three locations and three amplitudes) for its characterization.

In summary, the model selection based on 10-fold CV recovered a dense true distribution as correctly as RCV(n_v) did with a sparse one. However, a dense solution means that our fundamental hypothesis that the true model is sparse failed; hence it is better to look for other types of models that can be characterized by a low number of parameters. On the grounds of the results of this study, the suggested final algorithm for the analysis of unknown kinetics hypothesized to be of first order is Algorithm 3.

Algorithm 3

---

1. Execute a 2D BO optimization on a wide range of both λ and ω, applying 10-fold CV with the GENP.
2. Fix the optimal value of λ obtained in Step 1 and execute a BO on ω only.
3. If the value of ω is not low, the expected solution is not sparse. Go to Step 6.
4. The expected solution is sparse. Execute Algorithm 2.
5. Return.
6. The algorithm reached the limits of its valid range: the kinetics cannot be described by first-order reactions. It is still worthwhile to execute Steps 1 to 4 of Algorithm 1. Depending on the shape of the solution obtained, try to analyze the data with different sorts of models.

It is beyond the scope of this study to investigate how low the value of ω should generally be in Step 3 of this algorithm to ensure sparsity. As a guideline, Fig 3B and Fig 6B indicate that ω = 10^{-4} is a safe upper limit.

Possible extensions

Our results can be extended in many directions. The most obvious extensions are the cases when an eigenvalue of rate matrix $\mathbf{K}$ is degenerate with multiplicity of n, leading to a term of

$$At^{n-1}e^{-kt} \tag{12}$$

appearing in the solution of the rate equations. In the special case of $A = k^n/(n-1)!$ formula (12) is called Erlang distribution, which – independently of first-order reactions – can be used e.g. for modeling intracellular processes with molecular memory [73]. The simplest first-order reaction scheme leading to formula (12) with $n = 2$ is

$$A \xrightarrow{k} B \xrightarrow{k} C. \tag{13}$$

Is it in the problem or the solution?

To test Algorithm 3 for such a term, alone and mixed with true exponentials, we simulated data in the form of

$$te^{-\frac{t}{\tau_1}} + A(e^{-\frac{t}{\tau_2}} + e^{-\frac{t}{\tau_3}}) \tag{14}$$

with both zero and nonzero value of A in the presence of low and high noise levels. The solutions of the GENP with hyperparameters selected by RCV(n_v) and 10-fold CV are presented in Fig 12 for noise level $\sigma_{rel} = 10^{-7}$ and in S7 Fig for $\sigma_{rel} = 10^{-3}$. Strikingly, at low noise, the level of the regularization with both types of CVs is low (very low λ and medium ω), which leads to a relatively sparse solution. At the lowest regularization (10-fold CV, Fig 12 B), the solution is as expected for scheme (13): a negative spike followed by a positive one with some ringing at both

sides, and this characteristics remains with RCV(n_v) (Fig 12 A). The inclusion of pure exponential terms (Fig 12 C and D) results in the expected pattern. At high noise, as anticipated, regularization is higher, but the overall pattern remains unchanged (S7 Fig). The value of ω in no case approaches 1, indicating that Algorithm 3 correctly recognizes formula (14) as first-order kinetics.

Fig 12. Solution of the GENP obtained from data described by formula (14) at low noise. For (A) and (C), model selection was carried out by RCV(n_v), while for (B) and (D) by 10-fold CV. For (A) and (B), the value of A in the formula is 0, while for (C) and (D) its value is 20. $\tau_1 = 10^1$, $\tau_2 = 10^{-1}$, $\tau_3 = 10^3$. Noise level: $\sigma_{rel} = 10^{-7}$. λ and ω are the hyperparameters found by the BO process.

Other interesting directions of potential extensions are kinetic networks containing both first-order and other type of reactions. As a pilot study, we tested Algorithm 3 with simulated data of pure second-order kinetics and their mixture with first-order ones described by

$$\frac{C}{1 + C\frac{t}{\tau_1}} + A(e^{-\frac{t}{\tau_2}} + e^{-\frac{t}{\tau_3}}) \tag{15}$$

again, with both zero and nonzero value of A in the presence of low and high noise levels. The solutions of the GENP with hyperparameters selected by RCV(n_v) and 10-fold CV are presented in Fig 13 for noise level $\sigma_{rel} = 10^{-7}$ and in S8 Fig for $\sigma_{rel} = 10^{-3}$. As expected, 10-fold CV resulted in a value of ω close to 1 in every case, indicating that the overall kinetics are not of first order (Fig 13 and S8 Fig B and D). At low noise, the presence of the included exponential terms are manifested in narrow spikes, and the complete distribution clearly indicates the mixture of first-order and non-first-order reactions (Fig 13 D). At high noise, this pattern is less obvious (S8 Fig

D). Although the RCV(n_v) model selection resulted in a low value of ω, at low noise even this procedure provides similar patterns.

Fig 13. Solution of the GENP obtained from data described by formula (15) at low noise.

For (A) and (C), the model selection was carried out by RCV(n_v), while for (B) and (D) by 10-fold CV. For (A) and (B), the value of A in the formula is 0, while for (C) and (D) its value is 1. $C=8$, $\tau_1=10^1$, $\tau_2=10^{-1}$, $\tau_3=10^3$. Noise level: $\sigma_{rel}=10^{-7}$. λ and ω are the hyperparameters found by the BO process.

Conclusions

We find that group elastic net is a powerful and flexible method for the analysis of kinetic data. With properly selected hyperparameters λ and ω it provides a sparse recovery of kinetic parameters describing a system of first-order reactions, even of a complex scheme. Other values of the hyperparameters result in dense solutions, clearly indicating that the reaction is not of first order. We also find that the proper values of λ and ω can be found automatically by a machine-learning algorithm, utilizing a combination of the classical k-fold cross-validation and its novel RCV(n_v) version. Bayesian optimization has proved to be an ideal tool for solving the corresponding minimization problem. Our pilot study on the mixture of first-order and second-order reactions indicates that – at least at a low noise level – the presence of that mixture can be clearly recognized using Algorithm 3.

In this study we applied sophisticated statistical methods to build the machine-learning algorithms from the point of view of the experimentalist, irrespective of whether the strict theoretical conditions are satisfied for their justified application. For example, in the derivation of

RCV(n_v), Feng and Yu [48] supposed an additively separable penalty, while the group lasso term of GENP we applied does not have this property. Similarly, the same authors supposed that the support size of the solution is less than the number of data points, which was clearly not so in our calculations with a Gaussian true distribution. Nonetheless, the applied formulae worked well in practice. Yet, further theoretical studies are needed to theoretically justify their application.

Note that both the simulated and the experimental data applied in this study were obtained from the kinetics of a light-induced process. In fact, the most complex schemes of first-order reactions can be initiated by ultrashort light pulses on physical, chemical and biological systems. Typically, the initial steps of these schemes take place in electronic and vibrational excited states, and are frequently followed by relaxation phenomena in the ground state. The demand for the algorithms presented in this study is expected to soar due to the emerging ultrafast multidimensional infrared and electronic spectroscopic techniques [13, 74], as they are able to provide large and complex datasets. The ability of our algorithm to automatically recognize nonexponential decays could be utilized in the studies of nonadiabatic systems, where the relaxation of the electronically excited state is strongly coupled to coherent nuclear wavepacket motions [75].

In addition to the analysis of experimental data, the presented methods can be used to design experiments aimed at proving hypothetical models. Simulations with different noise levels – as presented on bR (S2 Table) – can predict the signal-to-noise ratio required to demonstrate the existence of non-dominating kinetic components.

Supporting information

S1 Table. Matrix of first-order microscopic rate constants corresponding to the bR photocycle model presented in Scheme 2 of the main text.

S1 Fig. Spectral and kinetic properties of the components presented in S1 Table.

(A) Absorption spectra (B) Difference spectra obtained by subtracting the spectrum of the bR state. (C) The corresponding kinetics calculated from the microscopic rate constants.

S2 Fig. Simulation of realistic, wavelength- and time-dependent noise for an absorption kinetic experiment on a bR sample.

(A) Blue line: Spectral distribution of the measuring beam filtered by the bR sample. Red line: the distribution of the noise level of the blue intensity spectrum, proportional to the inverse of its square root. (B) The level of noise in the different time segments at a selected wavelength. (See Methods of the main text for details).

S3 Fig. Construction of kinetic data based on the Arrhenius equation with distributed activation energy.

(A) The supposed rate constant (red) and distribution (blue) over the activation energy. (B) The true distribution of the time constant calculated from the data presented in (A). The resulted kinetics is presented in Fig 8 of the main text (blue line).

S2 Table. The predicted time constants (τ) and amplitudes (A) obtained at different noise levels, compared to the true values. The prediction was calculated by Algorithm 1 on the simulated data at different noise levels (σ_{rel}). λ and ω are the selected hyperparameters, MSE refers to the mean square error of the fit.

S4 Fig. Detailed results of Algorithm 2 on the simulated data presented in Fig 4 of the main text.

Wavelength: 416 nm. (A) Distribution over time constants. (B) Simulated kinetics (blue) and fit (red) by the distribution presented in (A). (C) Residual of the fit presented in (B). (D) Residual of the correcting exponential fit with 8 components remaining after discretization of the data, neglecting the components with amplitudes less than 5% of the maximum amplitude value. (E) Residual of the correcting exponential fit by keeping all 17 components obtained by the discretization.

S5 Fig. DADS obtained from Algorithm 2 by keeping all 17 components of the discretization.

Noise level: $\sigma_{rel} = 10^{-7}$.

S3 Table. The predicted time constants (τ) and amplitudes (A) obtained with realistic, wavelength- and time-dependent noise compared to the true values. For details see the legend of S2 Table.

S6 Fig. Detailed results of Algorithm 2 on the experimental FAD fluorescence kinetic data presented in Fig 7 of the main text.

Wavelength: 520 nm. (A) Distribution over time constants. (B) Experimental kinetics (blue) and fit (red) by the distribution presented in (A). (C) Residual of the fit presented in (B). (D) Residual of the correcting exponential fit.

S7 Fig. Solution of the GENP obtained from data described by formula (14) of the main text at high noise.

Noise level: $\sigma_{rel} = 10^{-3}$. For details see the caption of Fig 12 of the main text.

S8 Fig. Solution of the GENP obtained from data described by formula (15) at high noise.

Noise level: $\sigma_{rel} = 10^{-3}$. For details see the caption of Fig 13 of the main text.

References

1. Grossweiner LI. The study of labile states of biological molecules with flash photolysis. Adv Radiat Biol. 1966;2:83-133. doi: 10.1016/B978-1-4832-3121-1.50007-4.
2. Austin RH, Beeson KW, Eisenstein L, Frauenfelder H, Gunsalus IC. Dynamics of ligand binding to myoglobin. Biochemistry. 1975;14(24):5355-73. PubMed PMID: ISI:A1975AX94000021.
3. Váró G, Lanyi JK. Kinetic and spectroscopic evidence for an irreversible step between deprotonation and reprotonation of the Schiff base in the bacteriorhodopsin photocycle. Biochemistry. 1991;30(20):5008-15. doi: 10.1021/bi00234a024.
4. Nagle JF, Zimányi L, Lanyi JK. Testing BR photocycle kinetics. Biophys J. 1995;68(4):1490-9. doi: 10.1016/S0006-3495(95)80321-8.
5. Balashov SP, Lu M, Imasheva ES, Govindjee R, Ebrey TG, Othersen B, et al. The proton release group of bacteriorhodopsin controls the rate of the final step of its photocycle at low pH. Biochemistry. 1999;38(7):2026-39. doi: 10.1021/bi981926a.
6. van Stokkum IHM, Lozier RH. Target analysis of the bacteriorhodopsin photocycle using a spectrotemporal model. J Phys Chem B. 2002;106(13):3477-85. doi: 10.1021/jp0127723.
7. Zimányi L. Analysis of the bacteriorhodopsin photocycle by singular value decomposition with self-modeling: a critical evaluation using realistic simulated data. J Phys Chem B. 2004;108(13):4199-209. doi: 10.1021/jp0364809.
8. Lorenz-Fonfria VA, Saita M, Lazarova T, Schlesinger R, Heberle J. pH-sensitive vibrational probe reveals a cytoplasmic protonated cluster in bacteriorhodopsin. Proc Natl Acad Sci U S A. 2017;114(51):E10909-E18. doi: 10.1073/pnas.1707993114.
9. Pal SK, Peon J, Zewail AH. Biological water at the protein surface: Dynamical solvation probed directly with femtosecond resolution. Proc Natl Acad Sci U S A. 2002;99(4):1763-8. doi: 10.1073/pnas.042697899. PubMed PMID: ISI:000174031100005.
10. Ruhman S, Hou B, Friedman N, Ottolenghi M, Sheves M. Following evolution of bacteriorhodopsin in its reactive excited state via stimulated emission pumping. J Am Chem Soc. 2002;124(30):8854-8. doi: 10.1021/ja026426q.
