# Supplementary material for: Machine-learning model selection and parameter estimation from kinetic data of complex first-order reaction systems
Source: PLoS One. 2021 Aug 9;16(8):e0255675. doi: 10.1371/journal.pone.0255675 (PMC8352076; doi:10.1371/journal.pone.0255675)
Supplement: Attachment — Submitted filename: Response to reviewers.pdf [file pone.0255675.s017.pdf]

Reviewer #1:

1. In the new Section from line 739-751 there is a conflicting notation in "A" which is used both as a species in (13) and as an algebraic parameter in Eq (12) - please change one of these.

Author's reply:

The notations in (13) were changed.

Reviewer #1:

2. The authors state that in response to query 5 that "As we argue in the revised version, however, the suggested consideration of the intrinsic noise due to the small size of the reaction system in the framework of NLA [sic] is impossible for real experimental data on the kinetics of the bR photocycle for which our simulations are presented. This is due to the fact that the noise level originating from a realistic measuring procedure is by more than four orders of magnitude higher". Could the authors please provide references in support of this statement?

Author's reply:

The requested references were added in line 602.

Reviewer #2:

The original manuscript required many grammatical corrections as indicated with my previous review. The authors have dealt with these diligently. Just a few minor corrections are needed in the revision.

Author reply:

Many thanks for the further corrections of the Reviewer, all of them were accepted, and the manuscript text was amended accordingly.
